# Lay-delivered talk therapies for adults affected by humanitarian crises in low- and middle-income countries

**DOI:** 10.1186/s13031-021-00363-8

**Published:** 2021-04-23

**Authors:** Grace K. Ryan, Andreas Bauer, Tarik Endale, Onaiza Qureshi, Asmae Doukani, Arlinda Cerga-Pashoja, Savvy K. Brar, Julian Eaton, Judith K. Bass

**Affiliations:** 1 Department of Population Health, Faculty of Epidemiology and Population Health, London School of Hygiene and Tropical Medicine, London, UK; 2 Centre for Global Mental Health, London School of Hygiene and Tropical Medicine, Office 142A, Keppel Street, London, WC1E 7HT UK; 3 Department of Psychology, University of Bath, Bath, UK; 4 Department of Counseling and Clinical Psychology, Teachers College of Columbia University, New York City, New York USA; 5 Global Health Training, Public Health England, London, UK; 6 Division of Data, Analytics, Planning and Monitoring, UNICEF, New York City, New York USA; 7 Mental Health, CBM Global, Laudenbach, Germany; 8 Department of Mental Health, Johns Hopkins University, Baltimore, MD USA; 9 Center for Humanitarian Health, Johns Hopkins University, Baltimore, MD USA

**Keywords:** Global mental health, Mental health and psychosocial support, Psychotherapy, Humanitarian crises, Lay workers, Low- and middle-income countries

## Abstract

**Background:**

Published by the World Health Organization (WHO) and United Nations High Commissioner for Refugees (UNHCR) in 2015, the mental health Gap Action Programme Humanitarian Intervention Guide (mhGAP-HIG) recommends brief versions of structured psychological interventions for people experiencing symptoms of common mental disorders (CMDs). mhGAP-HIG acknowledges a growing body of evidence suggesting these interventions can be delivered by lay workers to people affected by humanitarian crises in low- and middle-income countries (LMICs). However, there has not yet been a systematic review and synthesis of this evidence. This paper reports the results of a systematic review of qualitative, quantitative, and mixed-methods studies assessing the implementation and/or effectiveness of talk therapies for CMDs when provided by lay workers in LMICs to adults who have survived or are currently living in humanitarian situations.

**Methods:**

Seven electronic databases were searched: MEDLINE, Embase, PsycINFO, PsycEXTRA, Global Health, Cochrane Library, and ClinicalTrials.gov. We also hand-searched the contents pages of three academic journals, reference lists of 30 systematic reviews, and online resource directories of two mental health networks. A preliminary list of included studies was circulated to topical experts for review, and all included studies were backward and forward searched. All titles, abstracts, and full-texts were independently double-screened. Quality appraisal and data extraction were carried out by a single reviewer and checked by a second reviewer, using standardised tools. Any disagreements were discussed and referred to a third reviewer as needed.

**Results:**

We identified 23 unique studies and carried out a narrative synthesis of patient and implementation outcome data. Every evaluation of the effectiveness of lay-delivered talk therapies for adults affected by humanitarian crises in LMICs showed some treatment effect for at least one CMD, and often multiple CMDs. Implementation research generally found these interventions to be acceptable, appropriate and feasible to implement, with good fidelity to manualised therapies.

**Conclusion:**

Although results are promising, particularly for individually-delivered talk therapies based on cognitive behavioural therapy techniques, there is a high degree of heterogeneity in this literature. We make several recommendations on how to improve the quality and generalisability of research on this topic, to facilitate further evidence synthesis.

**Trial registration:**

PROSPERO registration number: CRD42017058287.

**Supplementary Information:**

The online version contains supplementary material available at 10.1186/s13031-021-00363-8.

## Background

In the year 2021, a record-breaking one in every 33 people will need humanitarian assistance (up from one in 45 people in 2020) [1]. Emerging risks resulting from climate change, difficult-to-control infectious diseases, escalating political conflicts and a global economic recession will continue to pose serious threats, even after the worst of the COVID-19 pandemic is over [1, 2]. These trends have worrying implications for global mental health and well-being, which are now included in the United Nations (UN) 2030 Sustainable Development Agenda [3, 4]. Exposure to conflict, disasters, and other situations of extreme adversity increases the risk of developing common mental disorders (CMDs) such as depression, anxiety, and other stress-related conditions, as well as alcohol and substance use disorders [5–10]. Meanwhile, it can also worsen the severity of pre-existing mental health conditions [11]. A recent meta-analysis by Charlson et al. (2019) estimates a 22.1% point-prevalence for CMDs in conflict-affected populations, even after adjusting for comorbidity; over a third are moderate (4.0%) or severe cases (4.3%) of CMDs [6].

Addressing the mental health needs of populations affected by humanitarian crises is particularly challenging in low-resource settings. In low- and middle-income countries (LMICs), where mental health expenditure is typically below two dollars per capita [12], existing services are often heavily centralised, overstretched, and unable to cope with further disruptions to supply chains, destruction of infrastructure, loss of staff, and other common challenges encountered in crisis situations [11, 13, 14]. It is often where the needs are greatest that mental health services are least prepared to respond. For instance, Sub-Saharan Africa and South Asia are among the lowest ranking world regions on the Global Peace Index [15] and also have the fewest formally trained mental health workers [16].

Task-sharing, in which services are provided by non-specialists under the supervision of more highly trained providers [17], is an increasingly popular strategy to help overcome shortages of mental health specialists in these settings [18]. Recognising the need for more normative guidance on non-specialist mental health service delivery in humanitarian settings, the World Health Organisation (WHO) in collaboration with the UN High Commissioner for Refugees (UNHCR) issued a special version of the mental health Gap Action Programme (mhGAP) Intervention Guide in 2015 [19]. The mhGAP Humanitarian Intervention Guide (mhGAP-HIG) recommends brief versions of structured psychological interventions such as cognitive behavioural therapy (CBT) and interpersonal therapy (IPT) for people experiencing symptoms of CMDs in humanitarian settings [20]. The mhGAP-HIG also acknowledges a growing body of evidence suggesting that these interventions can be delivered by trained and supervised lay workers, a subset of non-specialists with no tertiary education or formal professional or paraprofessional certification in mental health [20]. However, there has not yet been a systematic review and synthesis of this evidence.

A 2013 Cochrane review covering a range of comparative study designs concluded that mental health interventions delivered by non-specialist health workers can improve outcomes for people with depression, post-traumatic stress disorder (PTSD), and alcohol-use disorder in LMICs [17]. A 2018 Cochrane review of trials conducted in LMICs affected by humanitarian crises found that psychological therapies can have a moderate to large effect in improving symptoms of depression, anxiety, and PTSD, though the quality of this evidence was considered low [21]. These and other similar reviews generally fail to differentiate between lay workers and other non-specialists such as general practitioners and nurses, who have substantially higher levels of education and medical training—and who are often exceptionally overstretched in crisis situations. Further, the ethical and logistical challenges of conducting experimental research in humanitarian settings [22] mean that reviews limited to comparative study designs capture only a fraction of the evidence generated in these contexts.

This is the first review to focus explicitly on the implementation and effectiveness of talk therapies delivered by lay workers to LMIC populations affected by humanitarian crises, despite the fact that lay workers have proven to be an essential human resource in these settings. The aims of this review are to investigate key characteristics of the interventions tested, the methods used to evaluate them, and the evidence generated to-date, in order to describe the current state of the research in this area.

## Methods

This paper presents results of a systematic review of qualitative, quantitative, and mixed-methods studies assessing the implementation and/or effectiveness of lay-delivered talk therapies for CMDs when provided to adults in LMICs who have survived or are currently living in crisis situations. Our methods were protocolised in accordance with the PRISMA checklist, registered on PROSPERO (CRD42017058287), and published in a peer-reviewed academic journal [23]. The iterative development of a Theory of Change also covered in our protocol will be described in a separate publication.

### Search strategy and selection criteria

A search strategy informed by previous reviews on related topics was developed, piloted, and refined in consultation with a qualified information specialist [17, 24]. Our search covered the following domains: LMICs, talk therapies, CMDs, lay workers, and humanitarian crises. Terms, headings, and syntax were adjusted for each of the electronic information sources consulted (see Additional File 5 for MEDLINE).

Six electronic databases were searched in May 2017: Ovid MEDLINE(R) (1946–2017); Embase (1974–2017); PsycINFO (1806–2017); PsycEXTRA (1908–2017); Global Health (1910–2017); Cochrane Library (all years). Between May and August 2017, we also hand-searched the content pages of three academic journals (*Conflict and Health*, *International Journal of Mental Health Systems*, and *World Psychiatry*), checked the reference lists of 30 published or in-press reviews on related topics [17, 22, 24–51], and searched the trial registry clinicaltrials.gov, plus resource directories of two mental health networks: mhinnovation.net/innovations and mhpss.net/resources. A preliminary list of 24 studies identified for inclusion was circulated to 15 experts in October 2019, to check for any missing manuscripts. Backward and forward searching of all included studies was carried out between February and March 2020.

Eligibility criteria are described further below. All titles, abstracts, and full-texts were independently double-screened, and every pair of screeners included at least one of the two lead reviewers (AB, GR). Any discrepancies were discussed between the two screeners and referred to a third screener as needed. In cases where there was insufficient information to make a final decision, corresponding authors were contacted. Authors were contacted at least twice at intervals of two weeks or longer before being marked non-responsive.

### Participants

We included studies with adults age 18 or over who have first-hand experience of a humanitarian crisis that occurred during their lifetime; for example, internally displaced persons (IDPs) and refugees, survivors of torture, and former soldiers.

We used Warren et al.’s (2015) definition of a humanitarian crisis as a disaster characterised by “a serious disruption of the functioning of a community or a society causing widespread human, material, economic or environmental losses which exceed the ability of the affected community or society to cope using its own resources, necessitating a request to national or international level for external assistance” (pp.2) [52]. We included both acute and protracted crises. In addition to consulting the list of protracted crises compiled by the Food and Agriculture Organization [53] as described in our original protocol, we also consulted the two sources used by the Organisation for Economic Co-operation and Development (OECD): (1) the World Bank’s annual Harmonized List of Fragile Situations; and (2) annual reports of the Fragile States Index (“High Alert” and “Very High Alert” Lists) [54–56]. In each case, we checked the available reports from the year closest to the text’s publication.

We excluded studies of interventions provided primarily to children or adolescents. Where studies covered ages above and below 18, we used the mean age of study participants receiving the intervention to assess eligibility; studies with mean age 18 or over were included. We also excluded studies with adults who were not alive at the time of the crisis (e.g., studies of interventions for the intergenerational transmission of trauma) and those who were incarcerated or serving in the military at the time of the study.

### Interventions

We included evidence-based talk therapies delivered through in-person dialogue with a trained lay worker, either one-to-one or in a group format, for the treatment of CMDs.

We adopted the definition of lay worker proposed by Lewin et al. (2005): “Any health worker carrying out functions related to health-care delivery; trained in some way in the context of the intervention; and having no formal professional or para-professional certificated or degreed tertiary education” (pp.7) [20]. We excluded teachers, as they often have tertiary education, and have not been classified as lay workers in previous reviews (e.g., van Ginneken et al. 2013) [17]. Although peers are not generally members of the health system before being recruited into delivery roles, we did include peer-delivered interventions, so long as peers met the educational criteria described above; hence, we refer to interventions included in this review as “lay-delivered”, as opposed to “lay health worker-delivered”.

We considered therapies to be evidence-based if they met one or more of the three criteria for “probably efficacious treatments” outlined by Chambless et al. (1998) [57, 58], as described in our review protocol [23]. Given challenges in differentiating between evidence-based psychotherapies and less structured psychosocial interventions based loosely on principles or techniques employed in these therapies [59], we also required the intervention to have been manualised at the time of study.

We included studies that expressly targeted one or more CMDs, even if participants did not have a confirmed diagnosis or were sub-threshold. This is in line with current thinking and advice on provision of psychotherapeutic interventions [60]. For the purposes of this review, CMDs comprised the following categories from the 2016 International Classification of Diseases that are most relevant to adults affected by humanitarian crises: depressive and other mood disorders (excluding manic episode and bipolar affective disorder); anxiety, phobic, dissociative, somatoform, obsessive-compulsive and other neurotic disorders; adjustment disorders and reactions to severe stress, including PTSD; and alcohol and substance use disorders.

To improve the specificity of our review, we excluded body psychotherapies and any other therapy that is not delivered primarily via face-to-face dialogue (e.g. Eye Movement Desensitization and Reprocessing [EMDR], Thought Field Therapy, self-help, computer- and phone-based interventions). Psychological First Aid and other psychoeducational, supportive, counselling and psychosocial interventions without a clearly defined, evidence-based psychotherapeutic component, were also excluded.

### Study characteristics

We included quantitative, qualitative and mixed-methods studies evaluating the implementation and/or effectiveness of relevant interventions. Studies were included regardless of whether they employed a comparative design, so long as they reported on one or more of the patient outcomes used by van Ginneken et al. (2013) [17] or implementation outcomes outlined by Proctor et al. (2011) [61], as described in our review protocol. We included only LMIC studies, based on the World Bank classification of country income status at the time of publication. We excluded study protocols, individual case reports, literature reviews, ecological studies, prevalence studies, and any other study design that did not meet the above criteria.

### Publication types

There were no restrictions on the year or language of publication, though our search terms were not optimised for languages other than English. Both grey and scholarly literature were considered. However, we excluded unpublished literature (e.g., incomplete studies and manuscripts under preparation).

### Data extraction, quality assessment and synthesis

We developed, piloted and refined a Microsoft Excel-based data extraction sheet, as described in our original protocol (see Additional Files 1 and 2 for full data extraction). This covered publication and study details, key features of the intervention, patient outcomes related to CMDs (improvement of symptoms, psychosocial functioning, disability) and implementation outcomes (acceptability, adoption, appropriateness, feasibility, fidelity, cost, penetration, sustainability). Reviewers extracted and then summarised any quantitative and/or qualitative data related to these outcomes. Data were extracted by one of the two lead reviewers and verified by a second reviewer. Any disagreements were discussed and referred to a third reviewer, as necessary.

Given that the number of studies identified for inclusion was substantially larger than originally anticipated, quality was not independently assessed by two reviewers as planned in our protocol. Instead, one of the two lead reviewers carried out quality assessment using either the Effective Public Health Practice Project (EPHPP) Quality Assessment Tool for Quantitative Studies [62], the Critical Appraisal Skills Programme (CASP) Qualitative Researcher Checklist [63], or a combination of the two for mixed-methods studies. The assessment was then checked by a second reviewer, and any disagreements were discussed and referred to a third reviewer as needed. We did not exclude studies from our synthesis on the basis of quality, as it is notoriously difficult to carry out gold-standard research in humanitarian settings [22].

Our protocol describes an intensive process for narrative synthesis adapted from guidance produced by Popay et al. (2006) for the Economic and Social Research Council UK Methods Programme [64], and by De Silva et al. (2014) for the application of Theory of Change to the UK Medical Research Council’s Framework for Complex Interventions [65]. These methods were developed with the end goal of producing a Theory of Change map for the delivery of talk therapies by lay workers to adults affected by humanitarian crises in LMICs, which will be reported in a separate publication. The present paper shares results of a more conventional synthesis involving tabulation of extracted data, followed by exploration of groupings and clusters within the data, carried out by the first author. We have elected to stage our synthesis in this manner to ensure timely dissemination of relevant information for key stakeholders contributing to WHO’s new area of research on scalable psychological interventions for communities affected by adversity [66].

## Results

**Fig. 1:**
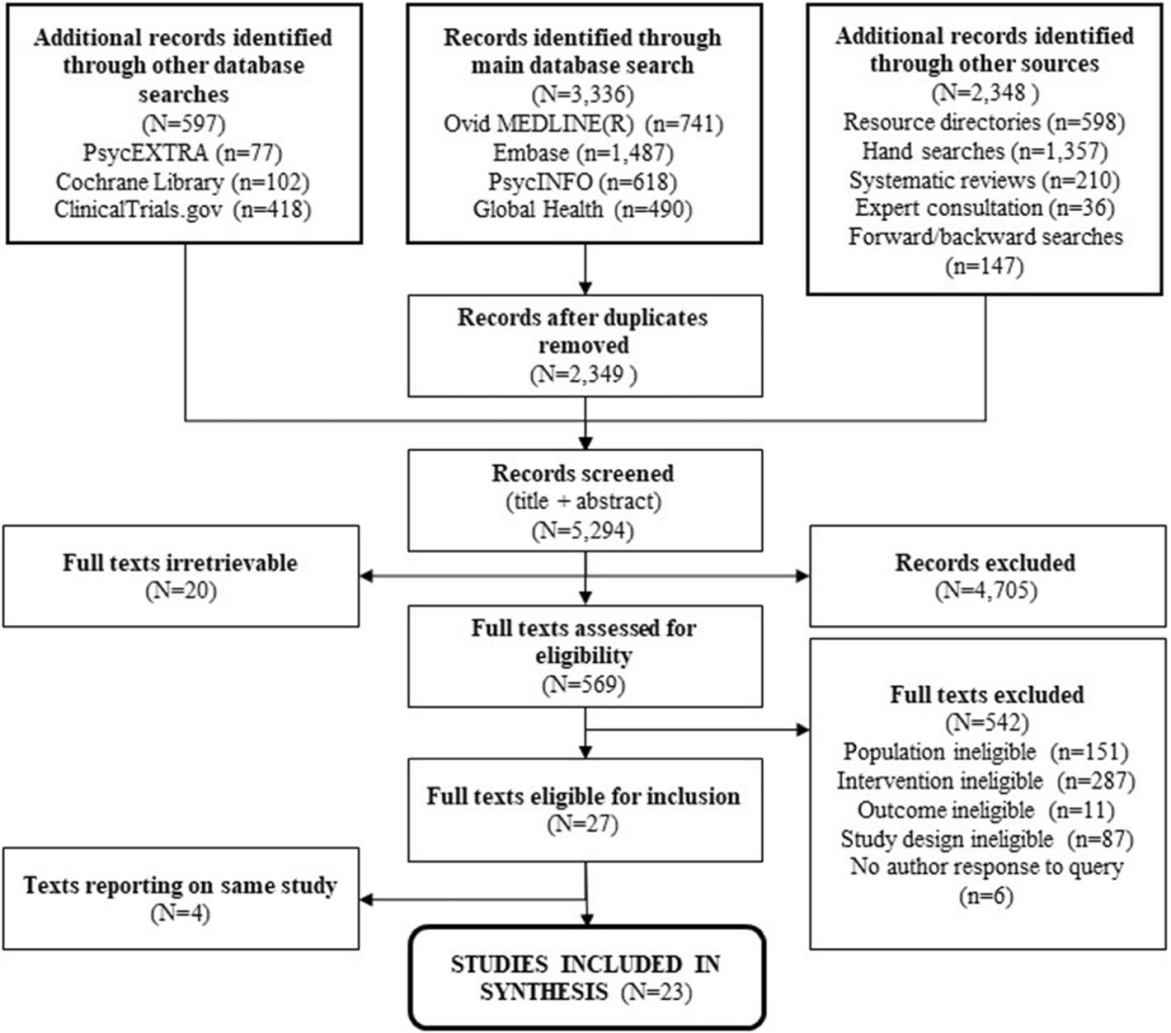
Flow Diagram

Our initial searches and expert consultation yielded 5294 unique records for title and abstract screening. Of the 589 that were identified for full text screening, 20 were irretrievable (e.g., titles/abstracts from conference proceedings, with no full texts available). The remaining 569 full texts were screened and 27 met inclusion criteria, representing 23 unique studies [see Fig. 1 for flow diagram].

### Included studies

A total of 27 texts were identified [67–93], dating back to 2005 and reporting on 23 original studies. The included texts reported on 10 studies evaluating patient outcomes [67–76, 90, 91], seven evaluating implementation outcomes [77–83, 92], and six evaluating both patient and implementation outcomes [84–89, 93]. Three were qualitative [78, 79, 83, 92], 15 were quantitative (including Rahman et al. 2007, which also reported qualitative results irrelevant to this review) [67, 68, 70–72, 74–77, 80, 84–87, 90, 91, 93, 94], and the remainder were multi-method studies [73, 81, 82, 88, 89]. Of those evaluating patient outcomes quantitatively, 12 used a comparison group [67–72, 74–76, 84, 88–91, 93]. Ten studies were carried out at the pilot stage [70, 73, 74, 79–81, 85, 87–89, 92], while the remainder were more formal evaluations of implementation or effectiveness [67, 68, 71, 72, 75–78, 82–84, 86, 90, 91, 93, 94]. Table 1 breaks these figures down by WHO region, providing a general picture of the research landscape. Additional File 1 provides further detail on study characteristics.

**Table 1:** Number of included studies, by WHO region and study type

	AFR	AMR	EMR	EUR	SEAR	WPR	Total
**Methods**							
Qualitative	1	1	1	0	0	0	**3**
Quantitative	7	1	4	0	3	0	**15**
Both	4	0	1	0	0	0	**5**
**Outcomes**							
Patient	6	1	3	0	0	0	**10**
Implementation	3	1	2	0	1	0	**7**
Both	3	0	1	0	2	0	**6**
**Stage**							
Pilot Stage	5	0	4	0	1	0	**10**
Full Evaluation	7	2	2	0	2	0	**13**

Studies were carried out in eight different countries, half from the WHO’s African region (“AFR”: Democratic Republic of the Congo [DRC] [69], Tanzania [81], Uganda [71, 72, 86, 87, 91], Zimbabwe [73–75, 82, 83, 88, 90]), a quarter from the Eastern Mediterranean region (“EMR”: Egypt [70], Pakistan [68, 76, 79, 80, 89, 92]), and the remainder from South-East Asia (“SEAR”: Thailand [77, 84, 85, 93]) and South America (“AMR”: Colombia [67, 78]). With the exception of Rahman et al. (2016a, 2016b) [76], studies from Zimbabwe and Pakistan did not explicitly target crisis-affected populations. However, they were published during periods of widespread, protracted crisis in each country (as indicated by a Fragile States Index score of 100 or higher, equivalent to “high” or “very high alert” status) [56].

Nine studies explicitly targeted displaced populations: one targeted internally displaced former child soldiers in Uganda [71]; six targeted refugees in Egypt [70], Tanzania [81], Thailand [77, 84, 85, 93] and Uganda [72, 91]; and two targeted both refugees and conflict-affected Ugandan nationals [86, 87]. Three studies targeted other survivors of violence, including sexual violence against women in DRC [69] and systemic violence against Afro-Colombians [67, 78]. The remaining studies targeted patient populations in fragile and conflict affected states [68, 73–76, 79, 80, 82, 83, 88–90, 92]; however, only two of these (Rahman et al. 2016a, Rahman et al. 2016b) were purposely sited in an area of the country directly affected by armed conflict [76, 89].

The therapies employed were mainly based on CBT techniques. These included a locally adapted, brief version of CBT (“Thinking Healthy”) [68, 79, 80, 92] as well as Cognitive Processing Therapy (CPT) [69, 81], Problem-Solving Therapy (PST) [73–75, 82, 83, 88, 90], Problem Management Plus (PM+) [76, 89], Narrative Exposure Therapy (NET) [71, 72, 86, 87, 91], and the Common Elements Treatment Approach (CETA), a transdiagnostic adaptation of CBT [67, 77, 78, 84, 85, 93]. Interpersonal Psychotherapy (IPT) was also used in one study [70].

Seven studies were of trauma-focussed therapies [69–72, 81, 86, 87, 91], though these studies generally recognised high co-morbidity between PTSD and other CMDs, for example by assessing depression as a secondary outcome. Nine studies were of depression-focussed therapies: five were specific to depression (four to maternal depression) [68, 74, 79, 80, 88, 92], and four targeted CMDs with a focus on depression [73, 75, 82, 83, 90]. Seven were transdiagnostic, explicitly targeting multiple CMDs [67, 76–78, 84, 85, 89, 93]. Only four studies evaluated interventions delivered in a group format (including one combining group and individual sessions) [69, 74, 79, 81, 92]. Table 2 outlines the different types of interventions and the populations they target, in each WHO region, and Additional File 2 provides further detail on the interventions and their implementation.

**Table 2:** Number of included studies, by region and intervention type

	AFR		AMR		EMR		EUR		SEAR		WPR		Total	
**Talk Therapy**														
IPT	0		0		1		0		0		0		**1**	
CBT-Based	12		2		5		0		3		0		**22**	
CETA		0		2		0		0		3		0		**5**
CPT		2		0		0		0		0		0		**2**
NET		4		0		0		0		0		0		**4**
PM+		0		0		2		0		0		0		**2**
PST		6		0		0		0		0		0		**6**
THP		0		0		3		0		0		0		**3**
**Therapy Focus**														
Transdiagnostic	0		2		2		0		3		0		**7**	
Focal	12		0		4		0		0		0		**16**	
Depression		6		0		3		0		0		0		**9**
Trauma		6		0		1		0		0		0		**7**
**Delivery Mode**														
Individual	9		2		5		0		3		0		**19**	
Group	3		0		0		0		0		0		**3**	
Both	0		0		1		0		0		0		**1**	
**Target Population**														
Survivors of Violence	1		2		0		0		0		0		**3**	
Displaced	5		0		1		0		3		0		**9**	
Internally Displaced		1		0		0		0		0		0		**1**
Refugees		2		0		1		0		3		0		**6**
Both		2		0		0		0		0		0		**2**
Patient Populations	6		0		5		0		0		0		**11**	
Perinatal/postnatal		1		0		3		0		0		0		**4**
People living with HIV		2		0		0		0		0		0		**2**
Other primary care attenders		3		0		2		0		0		0		**4**

### Excluded studies

Among the excluded studies, there were several borderline and other notable cases worth mentioning, as they offer relevant insight into key issues, namely: (1) the distinction between psychosocial counselling and psychotherapy; (2) age categories and youth-focussed interventions; (3) definitions of lay workers; (4) the distinction between crisis and adversity; and (5) controversial and potentially deleterious interventions. Reviewers seeking to replicate our methods would need to be aware of these issues and how they factored into screening decisions.

First, screeners noted it was often quite challenging to differentiate between evidence-based psychotherapies and the many counselling interventions drawing on psychotherapeutic approaches commonly used in humanitarian settings. For instance, the non-governmental organisation Médecins Sans Frontières (MSF) uses a manualised counselling intervention “based on principles derived from brief trauma-focused therapy and techniques from cognitive behavioural therapy” (Shanks et al. 2013), which has been delivered by lay health counsellors and evaluated in LMICs affected by humanitarian crises [14, 95]. Shanks et al. (2013) are explicit that MSF counselling does not constitute psychotherapy, but often this distinction is not made in-text, requiring further literature review and expert consultation.

Second, as researchers and implementers develop more sophisticated and locally appropriate definitions of youth, it becomes more challenging to differentiate between adult and adolescent mental health interventions. For example, Betancourt et al.’s 2014 trial of the CBT-based Youth Readiness Intervention for war-affected youth (ages 15–24) in Sierra Leone was excluded on the basis of age, despite meeting other criteria. While many participants were 18 or older, the mean age of those receiving the intervention was just under 18 (17.8), and the focus on readying young people for education and employment opportunities was considered fairly age-specific by screeners.

Third, “lay” terminology is not applied consistently across studies, even of similar interventions and in similar parts of the world. To illustrate: both Rahman et al. (2016) and Khan et al. (2019) claim to use lay people for the delivery of Problem Management Plus (PM+) interventions in Pakistan [76, 96]. However, in Khan et al. (2019) these “lay helpers” are described as “graduates” with 16 years of education (indicating a university degree), resulting in exclusion, whereas Rahman et al. (2016) only require 12 years of education (a secondary school degree). Similarly, trials of CETA in Southern Iraq and Thailand are both described by Murray et al. (2019) as using “lay counsellors” [77, 93]; however, Weiss et al. (2015) explain that in Iraq, these are nurses or medics working in primary health centres [97].

Fourth, it is often unclear what counts as a humanitarian crisis-affected population. As observed in our initial scoping search, exposure to protracted crisis is not always described in-text, perhaps because these situations come to be accepted as the status quo [23]. Meanwhile, we identified several studies of relevant interventions carried out with populations affected by other kinds of adversity in LMICs that did not meet our inclusion criteria. Notably, the PM+ intervention tested in Pakistan has also been tested among women affected by gender-based violence and other forms of “urban adversity” in Nairobi slums [98]. However, this “adversity” was not explicitly linked to a humanitarian crisis, and Kenya has not been considered a “high alert” country for 10 years [56].

Finally, screeners were surprised to encounter studies of contentious interventions like Thought Field Therapy [99, 100] in the reference lists of relevant reviews [28, 33]. Although in 2016 Thought Field Therapy was added to the evidence-based practice registry of the United States Substance Abuse and Mental Health Services Administration, there is controversy as to whether it should qualify as an evidence-based psychotherapy, given its central premise “that psychopathology can be treated by removing blockages in invisible and unmeasurable energy fields” (Lilienfield 2019, pp. 245) [101]. For the purposes of this review, we decided to exclude Thought Field Therapy, mainly because those carrying out the screening deemed it was better described as a body psychotherapy than a talk therapy.

Studies of narrative, exposure and testimony therapies were perhaps more challenging to assess. These models typically involve detailed retelling of trauma experiences, which Lane et al. (2016) suggest could have negative effects, if carried out without a sound rationale and concrete guidelines [102]. Proponents of NET are quite explicit that its focus on the development of a life-long narrative as opposed to narrating a single traumatic event is intended to mitigate this risk [103], and it has proven effective in several randomised controlled trials (RCTs) [104]; therefore, we did consider it an evidence-based therapy. We excluded most studies of similar narrative and testimonial therapies for other reasons, such as delivery by a specialist (e.g., Cienfuegos and Monelli 1983) [105].

### Quality assessment

Six of the studies using quantitative methods had a global EPHPP rating of “strong” [67, 68, 71, 75, 76, 84]. Three were “moderate” [69, 72, 74], and 11 were “weak” [70, 73, 77, 80–82, 85–89]. Of the studies using qualitative methods, only one (Atif et al. 2016) reported on potential biases introduced by the researchers’ positionality [79]. In three studies, sample sizes for qualitative data collection were notably small (nine participants or fewer), without clear justification [73, 78, 88]. Three studies provided insufficient detail on their methods of analysis [73, 81, 89]. Additional Files 3 and 4 provide itemised results of EPHPP and CASP assessments.

### Synthesis

Every evaluation of effectiveness that we identified reported at least some treatment effect for one or more CMDs. Where measured, there was also evidence of improvement in functioning and disability (except for at the Quibdó site of the Colombian CETA trial [67]). Results were less consistent for trait anger and trauma-related guilt [70, 71]. Three trials reported that there were no negative or adverse effects linked to the intervention [70, 71, 84], though this was not made explicit in other texts. Due to the heterogeneity of the interventions, methods and outcomes reported, we have not performed a meta-analysis, and instead summarise the findings related to patient and implementation outcomes for each intervention below (with effect sizes and *p*-values, where available). Further details on study outcomes are reported in Additional File 1.

### Trauma-focussed therapies

#### Cognitive processing therapy

CPT was delivered in a group format to Congolese women with experience of sexual violence in DRC [69] and intimate partner violence in a Tanzanian refugee camp [81]. The cluster RCT carried out by Bass et al. (2013) reported large effect sizes and significant (*p* < 0.001) improvements in symptoms of depression and anxiety (*d* = 1.8 at end-of-treatment, *d =* 1.6 at 6 months), PTSD (*d =* 1.4 end-of-treatment, *d =* 1.3 6 months) and daily functioning (*d =* 1.1 end-of-treatment, *d =* 1.2 6 months), when comparing CPT to individual support from psychosocial assistants [69]. Multi-method formative research and piloting by Greene et al. (2019) in Tanzania concluded that the intervention was generally considered appropriate by participants and was feasible to implement; however, participants only attended two-thirds of the intervention’s eight sessions [81]. Interviews with low-attenders suggested that acceptability of the group-delivered intervention could be improved by homogenising the age composition of groups, as women were sometimes put into groups with elder relatives with whom they felt uncomfortable sharing information about violent relationships.

#### Interpersonal therapy

In a small pilot RCT in Egypt, individual IPT for Sudanese refugees resulted in a significantly greater (*p* < 0.01) improvement in PTSD symptoms compared to the waitlist control (IPT *d =* − 2.52, waitlist *d =* − 0.75) [70]. Improvements in depression (IPT *d* = − 2.38, waitlist *d* = − 0.47) and state anger (IPT *d =* − 1.21, waitlist *d* = − 0.41) were also significant (depression *p* = 0.04, state anger *p* = 0.01), though this was not the case for trait anger or the Conflict Tactics Scale measuring household violence. Implementation outcomes were not formally evaluated.

#### Narrative exposure therapy

Individual NET was tested in two separate three-arm RCTs in Northern Uganda [71, 72, 91] and two uncontrolled evaluations using routinely collected data in Central Uganda [86, 87], all with displaced persons. All four studies reported improvements in PTSD among those receiving NET; however, NET was not always superior to active controls [71, 72].

Neuner et al.’s (2008) trial with Rwandan and Somalian refugees reported no significant difference when comparing the effects of NET (*d* = 1.4) and trauma counselling (*d* = 1.5) on PTSD symptoms over a nine-month follow-up period [72]. Nevertheless, both interventions were significantly more effective than monitoring alone (NET *F*_1,112_ = 8.2, *p* = 0.005; trauma counselling *F*_1,112_ = 8.2, *p* = 0.008).

Ertl et al. (2011) found that a youth-adapted version of NET called “KIDNET” was superior to both academic catch-up (*F*_1,234.1_ = 5.21, *p* = 0.02) and wait-listing (*F*_1,228.3_ = 5.28, *p* = 0.02) for 12-month reduction of PTSD symptoms among internally displaced former child soldiers (mean age 18), with moderate effect sizes (*d* = 0.72 and 0.66, respectively) [71]. KIDNET was also superior to controls in improving functioning, with large effects in comparison to academic catch-up (*d* = 0.83, F_1, 235.8_ = 7.20, *p* = 0.008) and wait-listing (*d* = 0.97, *F*_1,229.5_ = 12.63, *p* < 0.001). For the reduction of trauma-related guilt, KIDNET was superior to wait-listing alone (*F*_1,224.5_ = 11.20, *p* < 0.001), and the effect size was large (*d* = 0.93); however, there was no statistically significant advantage to KIDNET when compared to the active control group (*F*_1,230.8_ = 1.94, *p* = 0.16). Further, there were no significant findings related to other secondary outcomes (depression, suicidal ideation).

Evaluating pre- and post-treatment outcomes of NET delivered by a non-governmental organisation in Central Uganda, both Kandah (2017) and Durant (2019) reported “clinically significant improvements” in PTSD (defined by Durant 2019 as mean PTSD checklist score reduction of 10 points or more) [86, 87]. However, only Kandah et al. (2017) reported on effect size and statistical significance (*d* = 1.8, *p* < 0.001). Both studies also assessed routinely collected process data on acceptability (via client satisfaction surveys) and fidelity (via session checklists completed by counsellors). All participants responded that NET had helped them either “a lot” (77.8% in 2017, 96.5% in 2019) or “a little” (22.2% in 2017, 3.5% in 2019), with none responding that it was unhelpful. Kandah (2017) also reported lower-than-expected attrition (30.2%) as an indicator of acceptability. More than 95% of NET core components were completed by counsellors in both studies (96.2% in 2017, 99.3% in 2019).

### Depression-focussed therapies

#### Problem-solving therapy

PST-based interventions have been tested for a range of different patient populations in Harare, Zimbabwe, notably through the Friendship Bench programme [73, 75, 82, 83]. We judged these populations as likely to have been exposed to a protracted crisis and included studies published in years when Zimbabwe’s Fragile States Index exceeded 100 [56]. It is worth noting that Friendship Bench was originally established in response to state violence that resulted in many people losing their homes and livelihoods in the area [73].

Chibanda et al. (2011) piloted the delivery of brief individual PST by lay workers on benches outside local primary care facilities. Six to eight weeks from baseline, primary care attenders who had participated in at least three sessions experienced a significant, 4.8-point reduction (*p* = 0.0087) in CMD symptoms measured via the Shona Symptom Questionnaire (SSQ) [73]. The RCT that followed showed significant (*p* < 0.001) improvements in SSQ score (adjusted mean difference − 4.86) as well as depression (− 6.36), anxiety (− 5.73), disability (− 6.08), and health-related quality of life outcomes (0.12), when comparing the Friendship Bench intervention to enhanced usual care at six-months [75]. A secondary analysis by Munetsi et al. (2018) concluded that benefits of the Friendship Bench intervention were similar for trial participants with suicidal ideation (adjusted mean difference − 5.38, *p* < 0.001) [90]. A multi-method evaluation by Abas et al. (2016) also reported that the level of acceptability was high, as indicated by the consistently high rate of attendance (mean 505 visits per year, 2010–2014), low turnover among lay workers (14 of 15 continued delivering PST for 4 years) and perceived benefits reported by clients, supervisor and lay workers in interviews and focus group discussions [82]. Another study involving in-depth interviews with lay health workers and clients with HIV highlighted the importance of using indigenous concepts in ensuring that the PST was locally appropriate and acceptable [83].

Recognising the role of depression as a driver of poor adherence to anti-retroviral therapy, Abas et al. (2018) piloted *Kuvhura Pfungwa*, an individual PST-based intervention delivered by a lay adherence counsellor in Harare [88]. At six months, there was a significant (*p* = 0.01) 4.7-point mean difference in depression between the intervention group and the enhanced usual care control group, after controlling for baseline score. However, the adjusted between-group difference (1.13, *p* = 0.284) was not significant for general symptoms of CMDs measured via the SSQ. Abas et al. (2018) concluded that acceptability was high, based on level of attendance (71% of participants completed all six sessions) and analysis of qualitative exit interviews. Review of a random sample of session recordings using a therapist competence checklist also suggested high (75%) fidelity.

While both Friendship Bench and *Kuvhura Pfungwa* offer individual PST, Chibanda et al. (2014) have also carried out a pilot trial of peer-facilitated group PST for postnatal depression in Harare [74]. At six weeks’ follow-up, symptoms of postnatal depression were significantly lower (*p* = 0.0097) in the PST group (mean score 8.22), compared to a control group receiving pharmacotherapy (mean score 10.7). There was no significant difference in mean scores between the two groups at baseline (17.3 PST, 17.9 pharmacotherapy, *p* = 0.581), indicating that change in symptoms was attributable to the intervention.

#### Thinking healthy programme

As in the case of Zimbabwe, we included Thinking Healthy studies from those years when Pakistan’s Fragile States Index exceeded 100 [56]. Rahman et al. (2008) carried out a cluster RCT of Thinking Healthy, which includes an individual, CBT-based talk therapy for perinatal depression, through a cluster RCT in Rawalpindi, Pakistan [68]. At both six and 12 months, depression (adjusted mean difference − 5.86 at 6 months, − 6.65 at 12 months), disability (− 1.80 at 6 months, − 2.88 at 12 months) and functioning outcomes (6.85 at 6 months, 8.27 at 12 months) were all significantly better in the intervention group compared to the control group receiving enhanced routine care (*p* < 0.001).

While Thinking Healthy was originally delivered by community health workers known as Lady Health Workers, they were often overburdened with competing responsibilities in primary care. More recent research has therefore focused on training new cadres of peer volunteers to deliver therapy. Atif et al. (2015, 2016) used qualitative methods to investigate the acceptability of peer delivery in a pilot study in Rawalpindi [79, 92]. Overall, peer volunteers were considered to be acceptable delivery agents by all key stakeholders, due to their personal attributes (such as being local, trustworthy, empathetic, and having similar experiences of motherhood), legitimacy, and credibility (perceived usefulness and cultural appropriateness of the intervention, linkages with primary health care system).

### Transdiagnostic therapies

#### Common elements treatment approach

Lay-delivered individual CETA has been tested with survivors of systemic violence in both Thailand and Colombia [67, 77, 78, 84, 85, 93]. Murray et al. (2014) first piloted CETA along the Thai-Burmese border, monitoring outcomes on a weekly basis over a four- to 15-week period [85]. Clinically significant changes (exceeding minimal threshold *z* = 1.96 on Reliable Change Index) were reported for depression in 81.3% of participants, PTSD in 68.8% of participants, and functioning in 37.5% of participants. Acceptability was measured in terms of treatment retention (100%). Fidelity was not systematically assessed; however, review of supervisors’ and trainers’ notes indicated “good fidelity to the model, as evidenced by movement between and completion of each component’s steps” (Murray et al. 2014, pp.9).

After the pilot, Bolton et al. (2014) carried out a waitlist RCT demonstrating significantly greater (*p* < 0.001) improvements in the intervention group across nearly all outcomes at 4 months’ follow-up: depression, anxiety, PTSD, functioning and aggression [84]. Adjusted effect sizes were moderate (aggression *d* = 0.58, anxiety *d* = 0.79, functioning *d* = 0.63) to large (depression *d* = 1.16, PTSD *d* = 1.19), except in the case of alcohol use, for which there was no effect. A secondary analysis by Murray et al. (2019a) found no evidence that the trajectory of change among CETA recipients varied according to personal characteristics such as age or gender [93]. Bolton et al. (2014) also reported on attrition as an indicator of acceptability, noting fewer losses to follow-up in the intervention arm (*n* = 34) compared to the control arm (*n* = 39). An analysis of session-level data from the same RCT examined the fidelity of CETA delivery through weekly Client Monitoring Forms, and reported 100% provider compliance to delivery of the intervention’s “core flow elements” [77].

In Colombia, a three-arm RCT compared CETA against a waitlist group receiving monthly monitoring and an active control group receiving a narrative community-based group therapy, for Afro-Colombian survivors of systemic violence [67]. However, Bonilla-Escobar et al. (2018) report results of the CETA-waitlist comparison, exclusively. At one of the two study sites (Quibdó), there was no evidence of effect for any outcome except for reduction in PTSD; the effect size was small (*d* = 0.31) and failed to reach statistical significance (*p* = 0.053). At the second site (Buenaventura), there were significant effects (*p* < 0.001) for all outcomes, including moderate effects on PTSD (*d* = 0.70) and functional impairment (*d* = 0.70), and large effects on depression (*d =* 1.03), anxiety (*d* = 0.80) and Total Mental Health Symptoms (*d* = 0.82). Bonilla-Escobar et al. (2018) note that despite geographic proximity and cultural and ethnic similarities between the two populations, Quibdó and Buenaventura differed in terms of social context (e.g., urbanization, conflict, poverty), the relative experience of the delivery agents, and the mean number of sessions attended—which may help to explain these results. Interviews with lay workers, supervisors and coordinators from the trial suggested that implementers perceived the intervention to be effective but had concerns regarding its acceptability [78]. They advised that further integration of traditional and cultural knowledge of Afro-descendent populations was needed.

#### Problem management plus

Individual PM+ has been tested in both pilot and full RCTs in a conflict-affected area of Peshawar, Pakistan [76]. Comparing PM+ to enhanced usual care, the pilot showed significant improvements in PTSD and functioning, with at least 90% reduction in geometric mean in the intervention group, after adjusting for baseline scores (geometric mean for PTSD 92%, *p* = 0.02; geometric mean for functioning 90%, *p* = 0.04). However, there was no significant change in psychological distress (the primary outcome) [89]. Qualitative methods are not described, but were purportedly used to assess acceptability and feasibility: “On qualitative evaluation of a sub-sample of participants and primary care staff, we found that the intervention was perceived as useful, and was successfully integrated into primary care centres” (Rahman et al. 2016a, pp. 183). The full trial that followed reported significantly (*p* < 0.001) greater improvements among participants receiving PM+, compared to those receiving enhanced usual care, across all outcomes: depression (effect size 0.87 at 1 week, 0.73 at 3 months), anxiety (0.88 at 1 week, 0.83 at 3 months), PTSD (0.54 at 1 week, 0.63 at 3 months), disability (0.72 at 1 week, 0.67 at 3 months) and problems for which help was sought (1-week assessment, 0.34 at 3 months) [76].

## Discussion

As described by Morina et al. (2017), “A major challenge for global mental health is to develop more low intensity interventions that can still achieve reasonable effect sizes but simultaneously provide cost-effective solutions to LMICs” (pp.17) [28]. Our review highlights substantial efforts in recent years to meet this challenge. Of the 27 texts identified (representing 23 studies), over half (15) were published after 2015 [67, 75–79, 81–83, 86–90, 93], when mhGAP-HIG was released [19]. Twelve reported the results of controlled studies [67–72, 74–76, 84, 88–91, 93], though none examined cost-effectiveness. It is worth noting that every evaluation of effectiveness showed some treatment effect, typically with moderate to large effect sizes, for one or more CMDs [67–76, 84–91, 93]. Further, implementation research generally found lay-delivered talk therapies to be acceptable, appropriate and feasible to implement [77–89, 92, 93], echoing findings from a recent review of implementation outcomes of non-specialist-delivered CBT in LMICs (though the authors note the variable quality of this research) [106]. Several studies also demonstrated that with appropriate supervision systems in place, lay workers can deliver manualised talk therapies with a high degree of fidelity [84–88].

An especially promising development in this area of research is the emergence of effective transdiagnostic psychotherapies (CETA, PM+) tested in crisis-affected populations [67, 76–78, 84, 85, 89, 93]. Transdiagnostic interventions are particularly valuable for task-sharing models involving lay workers. As Murray and Jordans (2016) note, many of the earlier studies in this area have been of more narrowly focussed therapies targeting one or two CMDs. Putting these into practice in populations with high rates of CMDs would require either multiple cadres of lay providers—each focusing on a specific condition—or that each lay provider has the capacity to provide multiple different therapies [107]. Either scenario would require substantially more training and supervision, which would be costly and potentially unfeasible in humanitarian settings.

While we anticipated in our protocol that substantial heterogeneity would preclude meta-analysis [23], it seems this might be possible in the near future— particularly for interventions based on CBT techniques, like CETA, CPT, NET, PM+ and THP, each of which was tested in at least one RCT rated “strong”, according to our EPHPP assessment [67, 68, 71, 75, 76, 84]. Virtually all of these interventions were delivered in populations directly affected by humanitarian crises (with the exception of THP [68, 79, 80, 92], which was implemented in a protracted crisis situation). We would recommend that this review be updated, with meta-analyses carried out to inform future revisions of mhGAP-HIG, and that researchers and funders take seriously the need to carry out more high-quality replication studies in the interim [108]. This will require more consistency in how lay workers are defined. As observed by Xiong et al. (2019) in their recent scoping review on paraprofessional psychological interventions for PTSD [109], often terms like “non-specialists” or “counsellors” are employed, with no specification of their education or training level.

Despite some encouraging findings, our review also indicates a number of gaps in terms of the quality and representativeness of the research carried out to-date. While 13 studies evaluated implementation outcomes [77–89, 92, 93], they mostly used qualitative or uncontrolled quantitative study designs with a high risk of bias, and none investigated adoption, cost, penetration or sustainability. All but one qualitative study [79] showed major oversights in terms of sampling and/or reporting. Most quantitative studies were rated “weak” on their handling of confounding [70, 73, 74, 77, 80–82, 85–89], only two were “strong” on blinding [68, 75], and even some of the most highly rated RCTs faced challenges in applying representative recruitment strategies—often relying on referrals from local leaders, clinicians or non-governmental organisations [67, 76, 84]. More than half of the studies identified came from the WHO African region [69, 71–75, 81–83, 86–88, 90, 91], and none came from LMICs in the Western Pacific or European regions. While some study participants (for example in the PM+ trial [76]) were also exposed to disasters, the focus was generally on populations affected by conflict. Given that climate change and outbreaks of infectious diseases (e.g. Ebola Virus Disease [EVD], Coronavirus Disease [COVID-19]) are major impetuses behind recent calls to invest in scalable psychological interventions, more research is needed on lay-delivery in these contexts [110–112].

Group interventions may be of particular interest to humanitarian actors in LMICs seeking to drastically increase service coverage [107]. Unfortunately, there is not yet enough evidence to answer whether lay-delivered group therapies are as effective as individual therapies. We identified only three studies of therapies delivered in a group format (PST, CPT) [69, 74, 81] and one in a mixed format (THP) [79, 92], and all but one of these were still at the pilot stage [69]. However, it is notable that individual versions of two of these therapies (PST, THP) have also been evaluated in similar contexts [68, 73, 75, 76, 88–90]. Superiority trials investigating the cost-effectiveness of group- versus individually-delivered versions of these therapies would seem to be a logical next step to build a more robust evidence base.

The screening process for this review also highlighted concerns about the use of pseudoscientific, poorly standardised and ethically questionable psychotherapeutic approaches in the treatment of CMDs in LMICs affected by humanitarian crises. Other researchers have raised similar issues; for instance, Lipinksi et al. (2016) identified two studies involving potentially harmful techniques in their 2016 review of psychosocial interventions implemented in the aftermath of the Indian Ocean Tsunami [45]. Clearer guidance both on evidence-based psychotherapies and potentially harmful psychological interventions are needed [101, 113]. This will require researchers to also be more rigorous in describing their interventions, how they are delivered, and by whom; adapting the TiDier checklist for use with non-specialist psychological interventions could be a starting-point, to improve standardisation [114]. Further, the presence or absence of negative or adverse effects of these interventions should be more systematically investigated and reported, in order to assess their comparative risks and benefit. These and other recommendations are summarised in Panel 1, below.

**Table:** 

**Panel 1. Summary of Recommendations for LMIC Research on Lay-Delivered Talk Therapies for Adults Affected by Humanitarian Crises**	
1. **Research a wider range of populations and settings**	
• More regionally representative research, particularly in the AMR, EUR and SEAR regions, and in a wider variety of AFR (beyond Uganda and Zimbabwe) and EMR countries (beyond Egypt and Pakistan).	
• More evidence on acute crisis situations.	
• More research on populations affected by disasters and disease outbreaks.	
2. **Apply a wider range and higher quality of research methods**	
• More qualitative and multi-method research.	
• Superiority trials comparing different modes of delivery (individual vs. group).	
• More research on cost-effectiveness. • More consideration of potential sources of bias in study design (e.g. selection bias, confounding, unblinding, etc.)	
3. **Improve guidance and reporting**	
• Standardise definitions of “lay workers”.	
• Produce guidance on evidence-based and potentially harmful psychological interventions for crisis-affected populations.	
• Apply TiDier checklist to improve reporting on key components of complex interventions (especially recruitment, training and supervision of lay workers). • Improve transparency of reporting on negative/adverse effects in intervention studies. • Improve quality of reporting on methods, particularly for qualitative research (e.g. methods of participant selection, approach to analysis, positionality of researcher, etc.)	

### Limitations

This was a challenging review to undertake, requiring substantial knowledge not just of psychological interventions and the geopolitical context of diverse settings, but also terminology related to lay worker arrangements in different countries. While we were able to augment our screening methods with several additional checks to improve reliability (e.g., checking for manualisation of therapies, reviewing OECD sources on fragility), we often had to rely on consultation with authors to confirm whether studies met our eligibility criteria. In addition to consultation with authors, expert review proved crucial to the screening process. For instance, one expert identified three papers that had mistakenly been included despite coming from an unaffected region of a conflict-affected country. While extensive consultation may be considered a strength of this review, it can also increase the potential for human error to influence our results.

The most obvious limitation of this review is that the initial database search was carried out in 2017. Given the fast pace of research in this area, the more recent forward searching and expert review are probably not sufficient to identify all relevant studies published since that time. For example, studies of Integrative Adapt Therapy (IAT) were excluded from this review, as it appeared that IAT had not yet met the Chambless et al. (1998) criteria at the time of screening [57, 58]. However, Tay et al.’s (2020) RCT in Malaysia recently reported that IAT was superior to CBT for the treatment of CMDs among Rohingya, Chin and Kachin refugees, suggesting this may no longer be the case [115]. As described above, we would recommend this review be updated to capture recent developments.

## Conclusion

While the mhGAP-HIG acknowledges the fast-growing evidence base for lay-delivered talk therapies in situations of adversity [19], this evidence has never before been synthesised—perhaps due to its heterogeneity, as well as challenges in defining and operationalising key concepts, such as what constitutes an evidence-based talk therapy, who counts as a “lay” worker, and which populations are considered to be affected by humanitarian crises. This presents challenges both to implementers and decision-makers searching for evidence on how to operationalise mhGAP-HIG, as well as researchers and funders seeking to pinpoint the knowledge gaps where further investigation is most crucial. We identified 23 LMIC studies evaluating patient and/or implementation outcomes, all of which reported promising results. Every evaluation of effectiveness showed some treatment effect, for at least one CMD. Individually-delivered talk therapies based on CBT techniques were the most commonly studied, yet even within this category there was significant heterogeneity in terms of the type and focus of therapy, the population targeted, and the methods of evaluation employed. Consequently, we do not draw any definitive conclusions regarding the implementation or effectiveness of lay-delivered talk therapies in crisis-affected populations at this time. Rather, this review makes several recommendations on how to improve the quality and generalisability of research in this area, and to help facilitate future evidence synthesis as it continues to develop.

## Supplementary Information

**Additional file 1.** “Key characteristics of included studies”.

**Additional file 2.** “Characteristics of interventions”.

**Additional file 3.** “Results of EPHPP Assessment”.

**Additional file 4.** “Results of CASP Assessment”.

**Additional file 5.** “Search Terms for Ovid MEDLINE(R)”.

## Data Availability

Not applicable.

## Abbreviations

AFR: WHO African Region
AMR: WHO Americas Region
CASP: Critical Appraisal Skills Programme
CBT: Cognitive Behavioural Therapy
CETA: Common Elements Treatment Approach
CMD: Common Mental Disorder
COVID-19: Coronavirus Disease 2019
CPT: Cognitive Processing Therapy
DRC: Democratic Republic of the Congo
EMDR: Eye Movement Desensitization and Reprocessing
EMR: WHO Eastern Mediterranean Region
EPHPP: Effective Public Health Practice Project
EUR: WHO European Region
EVD: Ebola Virus Disease
HIV: Human Immunodeficiency Virus
IDP: Internally Displaced Person
IPT: Interpersonal Therapy
KIDNET: Narrative Exposure Therapy for children and adolescents
LHW: Lay Health Worker
LMIC: Low- or Middle-Income Country
mhGAP: mental health Gap Action Programme
mhGAP-HIG: mhGAP Humanitarian Intervention Guide
MSF: Médecins Sans Frontières
NET: Narrative Exposure Therapy
OECD: Organisation for Economic Co-operation and Development
PM+: Problem Management Plus
PRISMA: Preferred Reporting Items for Systematic reviews and Meta-Analyses
PROSPERO: Prospective Register of Systematic Reviews
PST: Problem-Solving Therapy
PTSD: Post-Traumatic Stress Disorder
RCT: Randomised Controlled Trial
SEAR: WHO South-East Asian Region
SSQ: Shona Symptom Questionnaire
THP: Thinking Healthy Programme
TiDier: Template for Intervention Description and Replication
UK: United Kingdom
UN: United Nations
UNHCR: UN High Commissioner for Refugees
WHO: World Health Organisation
WPR: WHO Western Pacific Region

## References

[1] OCHA (2020). Global humanitarian overview 2021.

[2] Devi S (2021). 2021: a year of humanitarian need. Lancet.

[3] Izutsu T, Tsutsumi A, Minas H, Thornicroft G, Patel V, Ito A (2015). Mental health and wellbeing in the sustainable development goals. Lancet Psychiatry.

[4] Lund C, Brooke-Sumner C, Baingana F, Baron EC, Breuer E, Chandra P, Haushofer J, Herrman H, Jordans M, Kieling C, Medina-Mora ME, Morgan E, Omigbodun O, Tol W, Patel V, Saxena S (2018). Social determinants of mental disorders and the sustainable development goals: a systematic review of reviews. Lancet Psychiatry.

[5] Ventevogel P, van Ommeren M, Schilperoord M, Saxena S (2015). Improving mental health care in humanitarian emergencies. Bull World Health Org.

[6] Charlson F, van Ommeren M, Flaxman A, Cornett J, Whiteford H, Saxena S (2019). New WHO prevalence estimates of mental disorders in conflict settings: a systematic review and meta-analysis. Lancet.

[7] de Jong JT, Komproe IH, Van Ommeren M (2003). Common mental disorders in postconflict settings. Lancet.

[8] van Ommeren M, Saxena S, Saraceno B (2005). Aid after disasters. BMJ (Clinical research ed).

[9] Steel Z, Chey T, Silove D, Marnane C, Bryant RA, van Ommeren M (2009). Association of torture and other potentially traumatic events with mental health outcomes among populations exposed to mass conflict and displacement: a systematic review and meta-analysis. JAMA..

[10] Greene MC, Kane JC, Krawczyk N, Brown F, Murray L, Khoshnood K, Tol WA, Morina N, Nickerson A (2018). Alcohol and drug misuse interventions in conflict-affected populations. Mental health of refugee and conflict-affected populations: theory, research and clinical practice.

[11] WHO (2013). Building Back Better: Sustainable Mental Health Care after Emergencies.

[12] WHO (2015). World Mental Health Atlas 2014.

[13] Tol WA, Barbui C, Bisson J (2014). World Health Organization guidelines for management of acute stress, PTSD, and bereavement: key challenges on the road ahead. PLoS Medicine.

[14] Souza R, Yasuda S, Cristofani S (2009). Mental health treatment outcomes in a humanitarian emergency: a pilot model for the integration of mental health into primary care in Habilla, Darfur. Int J Mental Health Sys.

[15] IEP (2019). Global Peace Index 2019: Measuring peace in a complex world.

[16] WHO (2018). Mental health atlas 2017.

[17] van Ginneken N, Tharyan P, Lewin S, Rao GN, Meera SM, Pian J, Chandrashekar S, Patel V. Non-specialist health worker interventions for the care of mental, neurological and substance-abuse disorders in low- and middle-income countries. Cochrane Database Syst Rev. 2013;(11):CD009149. 10.1002/14651858.CD009149.pub2. 10.1002/14651858.CD009149.pub2 24249541

[18] Murray LK, Tol W, Jordans M, Sabir G, Amin AM, Bolton P, Bass J, Bonilla-Escobar FJ, Thornicroft G (2014). Dissemination and implementation of evidence based, mental health interventions in post conflict, low resource settings. Intervention (Amstelveen).

[19] WHO and UNHCR (2015). mhGAP Humanitarian Intervention Guide (mhGAP-HIG): Clinical management of mental, neurological and substance use conditions in humanitarian emergencies.

[20] Lewin SA, Dick J, Pond P, Zwarenstein M, Aja G, van Wyk B, Bosch-Capblanch X, Patrick M. Lay health workers in primary and community health care. Cochrane Database Syst Rev. 2005;(1):CD004015. 10.1002/14651858.CD004015.pub2. 10.1002/14651858.CD004015.pub2 15674924

[21] Purgato M, Gastaldon C, Papola D, van Ommeren M, Barbui C, Tol WA (2018). Psychological therapies for the treatment of mental disorders in low- and middle-income countries affected by humanitarian crises. Cochrane Database Syst Rev.

[22] Tol WA, Barbui C, Galappatti A, Silove D, Betancourt TS, Souza R, Golaz A, van Ommeren M (2011). Mental health and psychosocial support in humanitarian settings: linking practice and research. Lancet.

[23] Ryan GK, Bauer A, Bass JK, Eaton J (2018). Theory of change for the delivery of talking therapies by lay workers to survivors of humanitarian crises in low-income and middle-income countries: protocol of a systematic review. BMJ Open.

[24] Singla DR, Kohrt BA, Murray LK, Anand A, Chorpita BF, Patel V (2017). Psychological treatments for the world: lessons from low- and middle-income countries. Annu Rev Clin Psychol.

[25] Chowdhary N, Sikander S, Atif N, Singh N, Ahmad I, Fuhr DC, Rahman A, Patel V (2014). The content and delivery of psychological interventions for perinatal depression by non-specialist health workers in low and middle income countries: a systematic review. Best Pract Res Clin Obstet Gynaecol.

[26] Clarke K, King M, Prost A (2013). Psychosocial interventions for perinatal common mental disorders delivered by providers who are not mental health specialists in low-and middle-income countries: a systematic review and meta-analysis. PLoS Med.

[27] Rahman A, Fisher J, Bower P, Luchters S, Tran T, Yasamy MT, Saxena S, Waheed W (2013). Interventions for common perinatal mental disorders in women in low-and middle-income countries: a systematic review and meta-analysis. Bull World Health Organ.

[28] Morina N, Malek M, Nickerson A, Bryant RA (2017). Meta-analysis of interventions for posttraumatic stress disorder and depression in adult survivors of mass violence in low-and middle-income countries. J Depress Anxiety.

[29] Bangpan M, Dickson K, Felix L, Chiumento A (2017). The impact of mental health and psychosocial support interventions on people affected by humanitarian emergencies: a systematic review. Humanitarian Evidence Programme.

[30] van der Waerden JEB, Hoefnagels C, Hosman CMH (2011). Psychosocial preventive interventions to reduce depressive symptoms in low-SES women at risk: a meta-analysis. J Affect Disord.

[31] Cuijpers P, Waheed W, Stein DJ, Van‘t Hof E (2011). Psychological treatments for depression and anxiety disorders in low-and middle-income countries: a meta-analysis. Afr J Psychiatry.

[32] Huntley AL, Araya R, Salisbury C (2012). Group psychological therapies for depression in the community: systematic review and meta-analysis. Br J Psychiatry.

[33] Palic S, Elklit A (2011). Psychosocial treatment of posttraumatic stress disorder in adult refugees: a systematic review of prospective treatment outcome studies and a critique. J Affect Disord.

[34] Crumlish N, O'Rourke K (2010). A systematic review of treatments for post-traumatic stress disorder among refugees and asylum-seekers. J Nerv Ment Dis.

[35] Gwozdziewycz N, Mehl-Madrona L (2013). Meta-analysis of the use of narrative exposure therapy for the effects of trauma among refugee populations. Permanente J.

[36] Williams ME, Thompson SC (2011). The use of community-based interventions in reducing morbidity from the psychological impact of conflict-related trauma among refugee populations: a systematic review of the literature. J Immig Minor Health.

[37] de Jong K, Knipscheer J, Ford N, Kleber RJ (2014). The efficacy of psychosocial interventions for adults in contexts of on-going man-made violence. Health..

[38] Dossa NI, Hatem M (2012). Cognitive-behavioral therapy versus other PTSD psychotherapies as treatment for women victims of war-related violence: a systematic review. Sci World J.

[39] Tol WA, Stavrou V, Greene MC, Mergenthaler C, van Ommeren M, García Moreno C (2013). Sexual and gender-based violence in areas of armed conflict: a systematic review of mental health and psychosocial support interventions. Confl Heal.

[40] Patel N, Kellezi B, de C Williams AC. Psychological, social and welfare interventions for psychological health and well-being of torture survivors. Cochrame Database Syst Rev. 2014. 10.1002/14651858.CD009317.pub2. 10.1002/14651858.CD009317.pub2 PMC11026826 25386846

[41] Nicholl C, Thompson A (2004). The psychological treatment of Post traumatic stress disorder (PTSD) in adult refugees: a review of the current state of psychological therapies. J Ment Health.

[42] Cuijpers P, Karyotaki E, Reijnders M, Purgato M, Barbui C (2018). Psychotherapies for depression in low-and middle-income countries: a meta-analysis. World Psychiatry.

[43] Mutamba BB, Nv G, Paintain LS, Wandiembe S, Schellenberg D. Roles and effectiveness of lay community health workers in the prevention of mental, neurological and substance use disorders in low and middle income countries: a systematic review. BMC Health Serv Res. 2013;13(1). 10.1186/1472-6963-13-412. 10.1186/1472-6963-13-412 PMC3852794 24119375

[44] Bunn M, Goesel C, Kinet M, Ray F (2015). Group treatment for survivors of torture and severe violence: a literature review. Torture..

[45] Lipinski K, Liu LL, Wong PW (2016). The effectiveness of psychosocial interventions implemented after the Indian Ocean tsunami: a systematic review. Int J Soc Psychiatry.

[46] Patel V, Araya R, Chatterjee S, Chisholm D, Cohen A, de Silva M, Hosman C, McGuire H, Rojas G, van Ommeren M (2007). Treatment and prevention of mental disorders in low-income and middle-income countries. Lancet.

[47] Cuijpers P, Cristea IA, Karyotaki E, Reijnders M, Huibers MJH (2016). How effective are cognitive behavior therapies for major depression and anxiety disorders? A meta-analytic update of the evidence. World Psychiatry.

[48] Verhey R, Chibanda D, Brakarsh J, Seedat S (2016). Psychological interventions for post-traumatic stress disorder in people living with HIV in resource poor settings: a systematic review. Tropical Med Int Health.

[49] Wiley-Exley E (2007). Evaluations of community mental health care in low-and middle-income countries: a 10-year review of the literature. Soc Sci Med.

[50] De Silva MJ, Cooper S, Li HL, Lund C, Patel V (2013). Effect of psychosocial interventions on social functioning in depression and schizophrenia: meta-analysis. Br J Psychiatry.

[51] Weiss WM, Uguento AM, Mahmooth Z (2016). Mental health interventions and priorities for research for adult survivors of torture and systematic violence: a review of the literature. Torture.

[52] Warren E, Post N, Hossain M, Blanchet K, Roberts B (2015). Systematic review of the evidence on the effectiveness of sexual and reproductive health interventions in humanitarian crises. BMJ Open.

[53] UN FAO (2015). The state of food insecurity in the world.

[54] World Bank (2020). The World Bank Group’s harmonized list of fragile situations: frequently asked questions.

[55] World Bank (2020). The World Bank’s fragile, conflict and violence group annual harmonized list of fragile situations.

[56] Fund for Peace (2020). Fragile states index: publications and downloads.

[57] Chambless D, Baker M, Baucom D (1998). Update on empirically validated therapies, II. Clin Psychol.

[58] Chambless DL, Hollon SD (1998). Defining empirically supported therapies. J Consult Clin Psychol.

[59] Duncan BL, Reese RJ, Weiner IB (2012). Empirically supported treatments, evidence-based treatments, and evidence-based practice. Handbook of psychology.

[60] Dawson KS, Bryant RA, Harper M, Kuowei Tay A, Rahman A, Schafer A, van Ommeren M (2015). Problem management plus (PM+): a WHO transdiagnostic psychological intervention for common mental health problems. World Psychiatry.

[61] Proctor E, Silmere H, Raghavan R, Hovmand P, Aarons G, Bunger A, Griffey R, Hensley M (2011). Outcomes for implementation research: conceptual distinctions, measurement challenges, and research agenda. Adm Policy Ment Health Ment Health Serv Res.

[62] EPHPP (1998). Quality assessment tool for quantitative studies.

[63] CASP (2018). Critical Appraisal Skills Programme, CASP (Qualitative) Checklist.

[64] Popay J, Roberts H, Sowden A (2006). Guidance on the conduct of narrative synthesis in systematic reviews.

[65] De Silva MJ, Breuer E, Lee L (2014). Theory of change: a theory-driven approach to enhance the Medical Research Council's framework for complex interventions. Trials.

[66] WHO (2017). Scalable psychological interventions for people in communities affected by adversity: a new area of mental health and psychosocial work at WHO.

[67] Bonilla-Escobar FJ, Fandiño-Losada A, Martínez-Buitrago DM, Santaella-Tenorio J, Tobón-García D, Muñoz-Morales EJ, Escobar-Roldán ID, Babcock L, Duarte-Davidson E, Bass JK, Murray LK, Dorsey S, Gutierrez-Martinez MI, Bolton P (2018). A randomized controlled trial of a transdiagnostic cognitive-behavioral intervention for afro-descendants’ survivors of systemic violence in Colombia. PLoS One.

[68] Rahman A, Malik A, Sikander S, Roberts C, Creed F (2008). Cognitive behaviour therapy-based intervention by community health workers for mothers with depression and their infants in rural Pakistan: a cluster-randomised controlled trial. Lancet.

[69] Bass JK, Annan J, Murray SM (2013). Controlled trial of psychotherapy for congolese survivors of sexual violence. N Engl J Med.

[70] Meffert SM, Abdo AO, Alla OAA (2014). A pilot randomized controlled trial of interpersonal psychotherapy for Sudanese refugees in Cairo, Egypt. Psychol Trauma Theory Res Pract Policy.

[71] Ertl V, Pfeiffer A, Schauer E, Elbert T, Neuner F (2011). Community-implemented trauma therapy for former child soldiers in northern Uganda: a randomized controlled trial. JAMA..

[72] Neuner F, Onyut PL, Ertl V, Odenwald M, Schauer E, Elbert T (2008). Treatment of posttraumatic stress disorder by trained lay counselors in an African refugee settlement: a randomized controlled trial. J Consult Clin Psychol.

[73] Chibanda D, Mesu P, Kajawu L, Cowan F, Araya R, Abas MA (2011). Problem-solving therapy for depression and common mental disorders in Zimbabwe: piloting a task-shifting primary mental health care intervention in a population with a high prevalence of people living with HIV. BMC Public Health.

[74] Chibanda D, Shetty AK, Tshimanga M, Woelk G, Stranix-Chibanda L, Rusakaniko S (2014). Group problem-solving therapy for postnatal depression among HIV-positive and HIV-negative mothers in Zimbabwe. J Int Assoc Provider AIDS Care.

[75] Chibanda D, Weiss HA, Verhey R, Simms V, Munjoma R, Rusakaniko S, Chingono A, Munetsi E, Bere T, Manda E, Abas M, Araya R (2016). Effect of a primary care–based psychological intervention on symptoms of common mental disorders in Zimbabwe: a randomized clinical trial. JAMA..

[76] Rahman A, Hamdani SU, Awan NR, Bryant RA, Dawson KS, Khan MF, Azeemi MMUH, Akhtar P, Nazir H, Chiumento A, Sijbrandij M, Wang D, Farooq S, van Ommeren M (2016). Effect of a multicomponent behavioral intervention in adults impaired by psychological distress in a conflict-affected area of Pakistan: a randomized clinical trial. JAMA..

[77] Murray LK, Haroz EE, Pullmann MD, Dorsey S, Kane J, Augustinavicius J, et al. Under the hood: lay counsellor element use in a modular multi-problem transdiagnostic intervention in lower resource countries. Cognitive Behav Ther. 2019;12. 10.1017/S1754470X18000144. 10.1017/S1754470X18000144 PMC6567986 31205483

[78] Pacichana-Quinayáz SG, Osorio-Cuéllar GV, Bonilla-Escobar FJ, Fandiño-Losada A, Gutiérrez-Martínez MI (2016). Common elements treatment approach based on a cognitive behavioral intervention: implementation in the Colombian Pacific. Ciencia Saude Coletiva.

[79] Atif N, Lovell K, Husain N, Sikander S, Patel V, Rahman A (2016). Barefoot therapists: barriers and facilitators to delivering maternal mental health care through peer volunteers in Pakistan: a qualitative study. Int J Mental Health Syst.

[80] Rahman A (2007). Challenges and opportunities in developing a psychological intervention for perinatal depression in rural Pakistan–a multi-method study. Archiv Women Mental Health.

[81] Greene MC, Rees S, Likindikoki S, Bonz AG, Joscelyne A, Kaysen D, Nixon RDV, Njau T, Tankink MTA, Tiwari A, Ventevogel P, Mbwambo JKK, Tol WA (2019). Developing an integrated intervention to address intimate partner violence and psychological distress in Congolese refugee women in Tanzania. Confl Heal.

[82] Abas M, Bowers T, Manda E, Cooper S, Machando D, Verhey R, Lamech N, Araya R, Chibanda D (2016). ‘Opening up the mind’: problem-solving therapy delivered by female lay health workers to improve access to evidence-based care for depression and other common mental disorders through the friendship bench project in Zimbabwe. Int J Ment Heal Syst.

[83] Chibanda D, Cowan F, Verhey R, Machando D, Abas M, Lund C (2017). Lay health workers’ experience of delivering a problem solving therapy intervention for common mental disorders among people living with HIV: a qualitative study from Zimbabwe. Community Ment Health J.

[84] Bolton P, Lee C, Haroz EE, Murray L, Dorsey S, Robinson C, Ugueto AM, Bass J (2014). A Transdiagnostic community-based mental health treatment for comorbid disorders: development and outcomes of a randomized controlled trial among Burmese refugees in Thailand. PLoS Med.

[85] Murray LK, Dorsey S, Haroz E, Lee C, Alsiary MM, Haydary A, Weiss WM, Bolton P (2014). A common elements treatment approach for adult mental health problems in low- and middle-income countries. Cogn Behav Pract.

[86] Durant S (2019). Effectiveness of narrative exposure therapy peer counseling with African refugees and Ugandan nationals: an archival study. The George Washington University.

[87] Kandah CC (2017). The use of peer counselors and narrative exposure therapy with African refugees and Ugandan nationals: an evaluation of program feasibility and preliminary effectiveness. Rosalind Franklin University of Medicine and Science.

[88] Abas M, Nyamayaro P, Bere T, Saruchera E, Mothobi N, Simms V, Mangezi W, Macpherson K, Croome N, Magidson J, Makadzange A, Safren S, Chibanda D, O’Cleirigh C (2018). Feasibility and acceptability of a task-shifted intervention to enhance adherence to HIV medication and improve depression in people living with HIV in Zimbabwe, a low income country in sub-Saharan Africa. AIDS Behav.

[89] Rahman A, Riaz N, Dawson KS, Usman Hamdani S, Chiumento A, Sijbrandij M, Minhas F, Bryant RA, Saeed K, van Ommeren M, Farooq S (2016). Problem management plus (PM+): pilot trial of a WHO transdiagnostic psychological intervention in conflict-affected Pakistan. World Psychiatry.

[90] Munetsi E, Simms V, Dzapasi L, Chapoterera G, Goba N, Gumunyu T, Weiss HA, Verhey R, Abas M, Araya R, Chibanda D (2018). Trained lay health workers reduce common mental disorder symptoms of adults with suicidal ideation in Zimbabwe: a cohort study. BMC Public Health.

[91] Onyut PL (2005). Setting up mental health provision by building local capacity: the case of Nakivale: epidemiological and treatment outcomes. University of Konstanz.

[92] Atif N (2015). The acceptability of peer volunteers as delivery agents of a psychosocial intervention for perinatal depression in rural Pakistan: a qualitative study. The University of Manchester.

[93] Murray LK, Haroz E, Dorsey S, Kane J, Bolton PA, Pullmann MD. Understanding mechanisms of change: An unpacking study of the evidence-based common-elements treatment approach (CETA) in low and middle income countries. Behav Res Ther. 2019;130:103430. 10.1016/j.brat.2019.103430. 10.1016/j.brat.2019.103430 PMC8114793 31780251

[94] Bass JK, Annan J, McIvor Murray S, Kaysen D, Griffiths S, Cetinoglu T, Wachter K, Murray LK, Bolton PA (2013). Controlled trial of psychotherapy for Congolese survivors of sexual violence. N Engl J Med.

[95] Shanks L, Ariti C, Siddiqui MR, Pintaldi G, Venis S, de Jong K, Denault M (2013). Counselling in humanitarian settings: a retrospective analysis of 18 individual-focused non-specialised counselling programmes. Confl Heal.

[96] Khan MN, Hamdani SU, Chiumento A, Dawson K, Bryant RA, Sijbrandij M, Nazir H, Akhtar P, Masood A, Wang D, Wang E, Uddin I, van Ommeren M, Rahman A (2019). Evaluating feasibility and acceptability of a group WHO trans-diagnostic intervention for women with common mental disorders in rural Pakistan: a cluster randomised controlled feasibility trial. Epidemiology and Psychiatric Sciences.

[97] Weiss WM, Murray LK, Zangana GAS (2015). Community-based mental health treatments for survivors of torture and militant attacks in Southern Iraq: a randomized control trial. BMC Psychiatry..

[98] Bryant RA, Schafer A, Dawson KS, Anjuri D, Mulili C, Ndogoni L, Koyiet P, Sijbrandij M, Ulate J, Harper Shehadeh M, Hadzi-Pavlovic D, van Ommeren M (2017). Effectiveness of a brief behavioural intervention on psychological distress among women with a history of gender-based violence in urban Kenya: a randomised clinical trial. PLoS Med.

[99] Sakai CE, Connolly SM, Oas P (2010). Treatment of PTSD in Rwandan child genocide survivors using thought field therapy. Int J Emerg Mental Health.

[100] Folkes CE (2002). Thought field therapy and trauma recovery. Int J Emerg Ment Health..

[101] Lilienfeld SO (2019). What is “evidence” in psychotherapies?. World Psychiatry.

[102] Lane W, Myers KJ, Hill MC, Lane DE (2016). Utilizing narrative methodology in trauma treatment with Haitian earthquake survivors. J Loss Trauma.

[103] Schauer M, Schauer E, Martz E (2010). Trauma-focused public mental-health interventions: A paradigm shift in humanitarian assistance and aid work. (2010) Trauma rehabilitation after war and conflict: Community and individual perspectives (pp 389-428) xviii, 436 pp New York, NY, US: springer science + business media; US.

[104] Robjant K, Fazel M (2010). The emerging evidence for narrative exposure therapy: a review. Clin Psychol Rev.

[105] Cienfuegos AJ, Monelli C (1983). The testimony of political repression as a therapeutic instrument. Am J Orthop.

[106] Verhey IJ, Ryan GK, Scherer N, Magidson JF (2020). Implementation outcomes of cognitive behavioural therapy delivered by non-specialists for common mental disorders and substance-use disorders in low- and middle-income countries: a systematic review. Int J Ment Heal Syst.

[107] Murray L, Jordans M (2016). Rethinking the service delivery system of psychological interventions in low and middle income countries. BMC Psychiatry.

[108] Haroz E, Nguyen A, Lee C, Tol W, Fine S, Bolton P (2020). What works in psychosocial programming in humanitarian contexts in low- and middle-income countries: a systematic review of the evidence. Intervention.

[109] Xiong T, Wozney L, Olthuis J, Swati Singh R, McGrath P (2019). A scoping review of the role and training of paraprofessionals delivering psychological interventions for adults with Post-traumatic stress. J Dep Anxiety.

[110] Costello A, Abbas M, Allen A, Ball S, Bell S, Bellamy R, Friel S, Groce N, Johnson A, Kett M, Lee M, Levy C, Maslin M, McCoy D, McGuire B, Montgomery H, Napier D, Pagel C, Patel J, de Oliveira JAP, Redclift N, Rees H, Rogger D, Scott J, Stephenson J, Twigg J, Wolff J, Patterson C (2009). Managing the health effects of climate change: *Lancet* and University College London Institute for Global Health Commission. Lancet.

[111] Torales J, O’Higgins M, Castaldelli-Maia JM, Ventriglio A (2020). The outbreak of COVID-19 coronavirus and its impact on global mental health. Int J Soc Psychiatry..

[112] Mohammed A, Sheikh TL, Poggensee G, Nguku P, Olayinka A, Ohuabunwo C, Eaton J (2015). Mental health in emergency response: lessons from Ebola. Lancet Psychiatry.

[113] Lilienfeld SO (2007). Psychological treatments that cause harm. Perspect Psychol Sci.

[114] Hoffmann TC, Glasziou PP, Boutron I, Milne R, Perera R, Moher D, Altman DG, Barbour V, Macdonald H, Johnston M, Lamb SE, Dixon-Woods M, McCulloch P, Wyatt JC, Chan AW, Michie S (2014). Better reporting of interventions: template for intervention description and replication (TIDieR) checklist and guide. BMJ..

[115] Tay AK, Mung HK, Miah MAA, Balasundaram S, Ventevogel P, Badrudduza M, Khan S, Morgan K, Rees S, Mohsin M, Silove D (2020). An integrative adapt therapy for common mental health symptoms and adaptive stress amongst Rohingya, Chin, and Kachin refugees living in Malaysia: a randomized controlled trial. PLoS Med.

