# Supplementary material for: Lay-delivered talk therapies for adults affected by humanitarian crises in low- and middle-income countries
Source: Confl Health. 2021 Apr 23;15:30. doi: 10.1186/s13031-021-00363-8 (PMC8062937; doi:10.1186/s13031-021-00363-8)
Supplement: Supplementary file 1 — Additional file 1. “Key characteristics of included studies”. [file 13031_2021_363_MOESM1_ESM.docx]

| Additional File 1. Key characteristics of included studies | | | | | | | | | | |
| --- | --- | --- | --- | --- | --- | --- | --- | --- | --- | --- |
| **Country** | **Therapy type** | **Location and setting** | **Author (year)** | **Target population** | **Target condition** | **Study design** | **Sample** | **Client outcomes and instruments** | **Implementation outcomes and instruments** | **Evidence of effect** |
| ***AFR*** |  |  |  |  |  |  |  |  |  |  |
| Democratic Republic of Congo (DRC) | Cognitive Processing Therapy (Group) | Community settings in North Kivu (2 villages) and South Kivu (14 villages) provinces. | Bass et al. 2013 | Women survivors of sexual violence in conflict-affected region of DRC | Anxiety, depression, PTSD | 2-arm cluster-randomised controlled trial  **Outcome assessment:** End-of-treatment and 6 months  **Comparison group:** Villages receiving individual support from psychosocial assistants | **Intervention:** 7 villages (157 women)  **Comparison:** 8 villages (248 women) | **Anxiety/depression:** 25-item Hopkins Symptom Checklist  **Functional impairment:** locally developed scale (20 items) **PTSD:** 16-item Harvard Trauma Questionnaire | N/A | **Anxiety/depression:** Effect size *d* = 1.8 (*p* < 0.001) at end of treatment, *d* = 1.6 (p<0.001) at 6 months  **Functional impairment:** Effect size *d* = 1.1 (*p* < 0.001) at end of treatment, *d* = 1.2 (*p* < 0.001) at 6 months  **PTSD:** Effect size *d* = 1.4 (*p* < 0.001) at end of treatment, *d* = 1.3 (*p* < 0.001) at 6 months |
| Tanzania | “Nguvu” (based on Cognitive Processing Therapy) | Nyarugusu refugee camp, northwestern Tanzania | Greene et al. 2019 | Congolese women refugees with experience of intimate partner violence | Anxiety, depression, PTSD | Multi-method formative research and piloting, including exit interview involving 4-item Likert scales and open-ended questions | **Quantitative:** 60 Congolese women refugees with 12-month history of intimate partner violence **Qualitative:** 7 high attenders (6-8 sessions) and 10 low attenders (0-3 sessions) | N/A | **Acceptability:** Participants attended 66% of sessions. Suggestions to improve retention as an indicator of acceptability (e.g. by homogenising age composition of groups, providing advance communication r.e. session scheduling, etc.)  **Appropriateness:** Generally considered relevant and helpful by participants  **Feasibility:** Intervention deemed feasible to implement | N/A |
| Uganda | Narrative Exposure Therapy (“KidNET” adapted for youth in Ertl et al. 2011) | Community-based NGO, Ndejje, Kampala, Uganda | Durant 2019 | Conflict-affected Ugandan nationals and African refugees | PTSD | Uncontrolled before- and-after study (analysis of routinely collected process and outcome data) **Outcome assessment:** End-of-treatment | 58 NGO clients for whom complete client files could be located (of the 60 treatment completers among 150 who entered programme) | **PTSD:** 17-item PTSD Checklist, Civilian Version (PCL-C) | **Acceptability**: 96.5% reported NET had helped "a lot", 3.5% "a little"  **Fidelity**: 96.6% of client files indicated 100% treatment fidelity; 99.3% of NET core components completed across all sessions | **PTSD:** 100% of clients reported a "clinically significant" (10 points or greater) decrease in PCL-C score between pre- and post-intervention assessments. Mean reduction was 38.75 points. No test for association between pre- and post-intervention assessments |
|  |  |  | Kandah 2017 |  |  |  | 30 treatment completers (of the 43 who entered the programme) |  | **Acceptability:** 77.8% of treatment completers reported that NET helped “a lot", 22.2% "a little"; 30.2% attrition rate (below the 40% attrition anticipated)  **Fidelity:** 96.2% of NET core components completed across all sessions | **PTSD:** Statistically significant (*p* < 0.001) and large effect size (*d* = 1.8) among the 30 treatment completers, between pre-intervention PCL-C score (mean 65.5) and post-intervention (mean 45.9) |
|  |  | Nakivale refugee settlement, southern Uganda | Ertl et al. 2011 | Internally displaced former child soldiers | Depression, PTSD | Three-arm randomised controlled trial  **Outcome assessment:** 3,6 and 12 months  **Comparison groups:** Academic catch-up with supportive counselling, and waitlist | **Intervention:** 29 former child soldiers  **Comparison:** 56 former child soldiers (28 per group) | **Depression/suicidal ideation:** Mini International Neuropsychiatric Interview (MINI) modules A and C  **Functional impairment/PTSD/trauma-related guilt:** Clinician-administered PTSD Scale (CAPS) | N/A | **Depression:** Effect size (Cohen’s *d*) 0.14 at 12 months when compared to academic catch-up; 0.40 compared to waitlist. Not statistically significant (*p* > 0.05)  **Functioning:** Effect size 0.83 at 12 months when compared to academic catch-up; 0.97 compared to waitlist. No adjustment for confounding. NET significantly more effective than academic catch-up (*p* = 0.008) and wait-list (*p* < 0.001)  **PTSD:** Effect size 0.72 at 12 months when compared to academic catch-up; 0.66 compared to waitlist. NET significantly more effective than academic catch-up and wait-list (*p* = 0.02)  **Suicidal ideation:** Effect size 0.12 at 12 months when compared to academic catch-up; 0.42 compared to waitlist. Not statistically significant (*p* > 0.05)  **Trauma-related guilt:** Effect size 0.38 at 12 months when compared to academic catch-up; 0.93 compared to waitlist. NET significantly more effective than wait-list (*p* < 0.001) but not academic catch-up (*p* > 0.05) |
|  |  |  | Neuner et al. 2008 | Refugees from Rwanda and Somalia displaced by conflict | PTSD | 3-arm cluster-randomised controlled trial  **Outcome assessment:** 3,6 and 9 months (9-month expert follow-up)  **Comparison groups:** Trauma counselling and monitoring groups | **Intervention:** 111 refugees  **Comparison:** 166 refugees (111 trauma counselling, 55 monitoring group) | **PTSD:** PTSD Scale (PDS), Composite International Diagnostic Interview (CIDI) | N/A | **PTSD:**  PDS: Both trauma counselling *F* (1, 112) = 8.2 (*p* = 0.0008) and NET *F* (1, 112) = 8.2 (*p* = 0.005) superior to monitoring group over follow-up period. Cohen’s *d* 1.4 in NET, 1.5 in trauma counselling and 0.8 in monitoring group at follow-up. No significant difference between NET and trauma counselling.  CIDI: Both trauma counselling *x*^2^ (1, *n* = 53) = 5.7 (*p* = 0.017) and NET *x*^2^(1, *n* = 65) = 4.4 (*p* = 0.036) superior to the monitoring group at 9 months. No significant difference between NET and trauma counselling |
|  |  |  | Onyut 2005 |  |  |  |  |  |  |  |
| Zimbabwe | Problem-Solving Therapy (group version in Chibanda et al. 2014) | Primary care clinics in Mbare, township south of Harare (Edith Opperman clinic in Chibanda et al. 2017) | Abas et al. 2016 | Primary care attenders in high-HIV prevalence area | CMDs | Multi-method study including qualitative (focus group discussion, in-depth interviews) and quantitative (analysis of routinely collected process data) methods | **Focus groups:** 8-12 lay health workers per group (5 groups)  **In-depth interviews:** 1 supervisor, 5 lay health workers, 6 clients | N/A | **Acceptability:**  High level of acceptability indicated by consistency in annual visits to Friendship Bench (mean 505 per year, 2010-2014), low turnover among lay health workers (14 of 15 continued to deliver assessments and problem-solving over 4 years; 1 passed away) and perceived benefits expressed by interviewees (clients, supervisor and lay health workers) | N/A |
|  |  |  | Chibanda et al. 2011 | Primary care attenders in high HIV-prevalence area affected by Operation Murambatsvina | CMDs | Uncontrolled before-and-after study **Outcome assessment:** end-of-treatment (6-8 weeks from baseline) | 320 primary care attenders | **Symptoms of CMDs:** Shona Symptom Questionnaire (SSQ) | N/A | **Symptoms of CMDs:** Mean 4.8-point reduction pre-/post-intervention (*p* = 0.0087) for those attending 3-6 sessions. Effects increased with number of sessions attended |
|  |  |  | Chibanda et al. 2017 | People living with HIV | CMDs | Qualitative (in-depth semi-structured interviews) with content analysis | 7 lay health workers (over 4 years experience each), and 10 participants with HIV (received intervention within the previous 2 years) | N/A | **Acceptability:** Authors highlighted use of indigenous concepts and other themes emerging from interviews indicating that intervention was culturally-appropriate and acceptable | N/A |
|  |  | Parirenyatwa Hospital Family Care Centre (university-affiliated government HIV clinic), Harare | Abas et al. 2018 | People living with HIV | Depression | Multi-method pilot involving 2-armed individually randomised controlled trial, fidelity checking (random 15% sample of session recordings) and qualitative exit interviews. **Outcome assessment:** 6 months **Comparison:** Enhanced usual care from HIV counsellors | **Intervention:** 14 people living with HIV  **Comparison:** 18 | **Symptoms of CMDs:** Shona Symptom Questionnaire  **Depression:** 9-item Patient Health Questionnaire (PHQ-9) | **Acceptability:** High level of acceptability, as indicated by frequency of session attendance (mean number of sessions attended 5.2; 71% completed all 6 sessions) and analysis of exit interviews, which identified a number of perceived benefits  **Fidelity:** >80% for first 2 sessions. Fidelity to adherence component dropped by fourth session (<65%) but remained high for problem-solving therapy component (75%) | **Depression:** Difference of 4.7 points between intervention and comparison at follow-up, after controlling for baseline score (*p* = 0.01). In terms of clinical significance, in the intervention arm, the average depression score on the PHQ-9 fell from ‘moderate depression’ range to ‘no depression’ range. In the EUC arm, average depression score on the PHQ-9 also fell, but remained in the ‘mild depression’ range at follow-up  **Symptoms of CMDs:** Difference of 1.13 points between intervention and comparison at follow-up, after controlling for baseline score, but not significant (*p* = 0.284) |
|  |  | Primary care antenatal clinics in Chitungwiza, periurban area southeast of Harare | Chibanda et al. 2014 | Postnatal women (6-8 weeks after childbirth) in high HIV-prevalence area | Postnatal depression | 2-arm individually randomised controlled trial  **Outcome assessment:** 6 weeks **Comparison:** Pharmacotherapy (amitriptyline) | **Intervention:** 30 mothers **Comparison:** 28 mothers | **Postnatal depression:** Edinburgh Postnatal Depression Scale (EPDS) | N/A | **Postnatal depression:** Significant (*p* = 0.0097) difference in EPDS score between intervention (8.22, SD 3.6) and comparison group (10.7, SD 2.7) at 6-week follow-up. No significant difference (*p* = 0.581) between intervention (17.3, SD 3.7) and comparison group (17.9, SD 3.9) at baseline, indicating improvement attributable to intervention |
|  |  | Primary care clinics in Harare | Chibanda et al. 2016 | Primary care attenders in high HIV-prevalence areas | CMDs: anxiety, depression | 2-arm cluster randomised controlled trial  **Outcome assessment:** 6 months  **Comparison:** Enhanced usual care by primary care nurses, with additional supportive messaging/calls | **Intervention:** 12 clinics, approx. 25 attenders per clinic (*n* = 286); **Comparison:** 12 clinics, approx. 25 attenders per clinic (*n* = 287) | **Anxiety:**  Generalised Anxiety Disorder Scale (GAD-7)  **Depression:** Patient Health Questionnaire (PHQ-9)  **Functioning:** World Health Organisation Disability Assessment Schedule version 2.0 (WHODAS 2.0)  **Quality of life:** EuroQOL 5D (EQ-5D)  **Symptoms of CMDs:** Shona Symptom Questionnaire (SSQ-14) | N/A | **Anxiety:** Significant (*p* < 0.001) difference between intervention and comparison group at 6 months (-5.73, CI -6.61 to -4.85), after adjustment (including for baseline scores)  **Depression:** Significant (*p* < 0.001) difference between intervention and comparison group at 6 months (-6.36, CI -6.45 to -5.27), after adjustment (including for baseline scores)  **Functioning:** Significant (*p* < 0.001) difference between intervention and comparison group at 6 months (-6.08, CI -7.46 to -4.71), after adjustment (including for baseline scores)  **Quality of life:** Significant (*p* < 0.001) difference between intervention and comparison group at 6 months (0.12, CI 0.08 to 0.17), after adjustment (including for baseline scores)  **Symptoms of CMDs:** Significant (*p* < 0.001) difference between intervention and comparison group at 6 months (-4.86, CI -5.63 to -4.10), after adjustment (including for baseline scores) |
|  |  |  | Munetsi et al. 2018 |  |  | Secondary analysis of cluster-randomised controlled trial data from Chibanda et al. 2016 |  |  |  | **CMD symptoms:** Similar and statistically significant improvements in severity of CMD symptoms among intervention recipients reporting suicidal ideation (mean difference -5.38, CI -7.85 to -2.90, *p* < 0.001) compared to those not reporting suicidal ideation (mean difference -4.86, CI -5.68 to -4.04, *p* < 0.001) |
| ***AMR*** |  |  |  |  |  |  |  |  |  |  |
| Colombia | Common Elements Treatment Approach | 2 largest municipalities in the Colombia Pacific region: Buenaventura, Valle del Cauca province; Quibdo, Choco province | Bonilla-Escobar et al. 2018 | Afro-Colombian survivors of systemic violence | CMDs: Anxiety, depression, PTSD | 3-arm randomized controlled trial  **Outcome assessment:** 2 weeks after end-of-treatment (3-4 months from baseline for standby group)  **Comparison:** narrative community-based group therapy, standby group under monthly monitoring | 521 Afro-descendents with history of violent trauma (180 Buenaventura, 166 Quibdo)  **Intervention:** 175 (92 Buenaventura, 83 Quibdo)  **Comparison:** 171 standby group (88 Buenaventura, 83 Quibdo); 172 narrative group (89 Buenaventura, 83 Quibdo) | **Anxiety, Depression, PTSD, Total Mental Health Symptoms:**  Locally-validated 64-item Total Mental Health Symptoms survey, with sub-scales for depression, anxiety and PTSD symptoms (based on (1) Hopkins Symptom Checklist, (2) Harvard Trauma Questionnaire, (3) PTSD Checklist-Civilian Version, (4) qualitative questions on symptoms)  **Functional impairment:** Gender-specific functional impairment scale (12-item for women, 10-item for men) | N/A | **Anxiety:** Buenaventura: large effect size (*d* = 0.80); Quibdo: no significant effect  **Depression:** Buenaventura: large effect size (*d* = 1.03); Quidbo: no significant effect  **Functional impairment:** Buenaventura: moderate effect size (*d* = 0.70); Quibdo: no significant effect  **PTSD symptoms:** Buenaventura: moderate effect size (*d* = 0.70); Quibdo: small effect size (*d* = 0.31)  **Total Mental Health Symptoms:** Buenaventura: large effect size (*d* = 0.82); Quibdo: no significant effect |
|  |  |  | Pacichana-Quinayáz et al. 2016 |  |  | Qualitative study (in-depth interviews either in-person or via video-call with Lay Psychosocial Community Workers, supervisors and coordinators responsible for implementation of CETA) | 9 participants: 5 Lay Psychosocial Community Workers (3 Quibdo, 2 Buenaventura), 2 coordinators (1 Quibdo, 1 Buenaventura), cross-site supervisor and Heartland Alliance International Coordinator | N/A | **Acceptability:** Participants noted the need for further integration of traditional and cultural knowledge of Afro-descendent populations (e.g. in terms of identification of local symptoms, use of relevant language for communicating about CETA) | **Perceived effectiveness:** Interviewees noted improvements (sometimes as early as first 3 sessions) in: clients' anxiety, expressiveness, confidence, appearance ("better face"), thoughts about the future, concentration, interpersonal relationships, and feelings of anger, sadness and fear. They also observed that clients were better able to recognise and manage symptoms using strategies learned through CETA |
| ***EMR*** |  |  |  |  |  |  |  |  |  |  |
| Egypt | Interpersonal Therapy (Individual) | Community-based organisation (Ma’an Organisation) office, Cairo | Meffert et al. 2014 | Sudanese refugees displaced by conflict | Depression, PTSD | Pilot randomised-controlled trial  **Outcome assessment:** End-of-treatment  **Comparison group:** Waitlist control group | **Intervention:** 13 refugees  **Comparison:** 9 refugees | **Depression:** Beck Depression Index-II  **PTSD:** Harvard Trauma Questionnaire  **State and trait anger:** State-Trait Anger Inventory  **Violence:** Conflict Tactics Scale-Household Violence | N/A | **Depression:** Within-subject effect size *d* = -2.38 for IPT, *d* = -0.47 waitlist. Statistically significant difference in reduction of depression symptoms between IPT and wait-list (*p* = 0.04)  **PTSD:** Within-subject effect size *d* = -2.52 for IPT, *d* = -0.75 waitlist. Statistically significant difference in reduction of PTSD symptoms between IPT and waitlist (*p* < 0.01)  **State anger:** Within-subject effect size *d* = -1.21 for IPT, *d* = -0.41 waitlist. Statistically significant difference in reduction of state anger between IPT and waitlist (*p* = 0.01)  **Trait anger:** Within-subject effect size *d* = -1.43 for IPT, *d* = 0.32 waitlist. Difference between groups not significant (*p* = 0.32)  **Violence:** Within-subject effect size *d* = -0.84 for IPT, *d* = -0.53 waitlist. Difference between groups not significant (*p* = 0.62) |
| Pakistan | “Thinking Healthy” (Based on Cognitive Behaviour Therapy, individual and group) | Community settings in Banda and Jatha Hathiyal  union councils, Rawalpindi, Punjab province | Atif et al. 2015 | Women in the perinatal period | Perinatal depression | Qualitative (in-depth interviews, focus groups) | **Interviews:** 34 participants (21 mothers, 8 peer volunteers, 5 primary care staff)  **Focus groups:** 2 groups, 15 participants total (5 husbands, 10 mothers-in-law) | N/A | **Acceptability:**  Peer volunteers considered acceptable delivery agents by all key stakeholders, due to several key factors: personal attributes (such as being local, trustworthy, empathetic, and having similar experiences of motherhood), legitimacy and credibility (perceived usefulness and cultural appropriateness of the intervention, linkages with primary health care system) | N/A |
|  |  |  | Atif et al. 2016 |  |  |  |  |  |  |  |
|  |  | Primary care centres and surrounding communities in rural areas of Rawalpindi | Rahman 2007 | Women in the perinatal period | Perinatal depression | Multi-method formative research and evaluation. Relevant component of evaluation research comprised a retrospective questionnaire on client satisfaction | All 164 mothers participating in intervention | N/A | **Acceptability**:  95% of participants rated the intervention as “useful” (47%) or “very useful” (48%) on exit questionnaire measuring self-reported client satisfaction on 5-item Likert scale | N/A |
|  |  |  | Rahman et al. 2008 | Women in the perinatal period | Perinatal depression | 2-arm cluster-randomised controlled trial  **Outcome assessment:** 6 and 12 months  **Comparison group:** Union council clusters receiving enhanced routine care by Lady Health Workers | **Intervention:** 20 clusters (*n* = 463 pregnant women)  **Comparison:**  20 clusters per (*n* = 440 pregnant women) | **Depression:** (1) Diagnosis of major depression by psychiatrist as per DSM-IV criteria; (2) Severity according to Hamilton Depression Rating Scale  **Disability:** Brief Disability Questionnaire  **Functioning:** Global Assessment of Functioning Scale | N/A | **Depression:** 6-month adjusted odds ratio for diagnosis in intervention compared to control: 0.22 (95% CI 0.14-0.36, *p* < 0.0001); 12-month adjusted odds ratio 0.23 (95% CI 0.15-0.36, *p* < 0.0001). 6-month adjusted mean difference in severity of depression between intervention and control: -5.86 (95% CI -7.92 to -3.80, *p* < 0.0001); 12-month adjusted mean difference: -6.65 (95% CI -8.56 to -4.74, *p* < 0.0001)  **Disability:** 6-month adjusted mean difference in disability between intervention and control: -1.80 (95% CI -2.48 to -1.12, *p* < 0.0001); 12-month mean difference: -2.88 (95% CI -3.66 to -2.10)  **Functioning:** 6-month adjusted mean difference in functioning between intervention and control: 6.85 (95% CI 4.73 to 8.96, *p* < 0.0001); 12-month mean difference: 8.27 (95% CI 6.23 to 10.31, *p* < 0.0001) |
|  | Problem Management Plus | Primary care centres in Peshawar, Pakistan | Rahman et al. 2016a | Primary care attenders living in a conflict-affected areas | CMDs: Psychological distress, PTSD | 2-arm randomised controlled trial  **Outcome assessment:** “The study was conducted from March to May 2014” (pp.182); No further details  **Comparison group:** Enhanced usual care from primary care physician | **Intervention:** 30 primary care attenders  **Comparison:** 30 primary care attenders | **Functioning:** 12-item World Health Organisation Disability Assessment Schedule  (WHODAS  2.0)  **Psychological distress:** 12-item General Health Questionnaire (GHQ-12)  **PTSD:** PTSD Checklist for DSM-5 (PCL-5) | **Acceptability, Feasibility:** Qualitative methods are not described, but were purportedly used to assess acceptability and feasibility: “On qualitative evaluation of a sub‐sample of participants and primary care staff, we found that the intervention was perceived as useful, and was successfully integrated into primary care centres.” (pp.183) | **Functioning:** 90% reduction in geometric mean within the intervention group (95% CI  90.4%-91.7%, *p* = 0.04) after adjusting for baseline  **Psychological distress: “**There was no significant change in GHQ-12 scores” (pp.182); no further details  **PTSD:** 92% (95% CI 91.2%-92.3%, *p* = 0.02) reduction in geometric mean within the intervention group, after adjusting for baseline |
|  |  |  | Rahman et al. 2016b |  | CMDs: Anxiety, depression, PTSD | 2-arm randomised controlled trial  **Outcome assessment:** 1 week and 3 months  **Comparison group:** Enhanced usual care from primary care physicians | **Intervention:** 172 primary care attenders **Comparison:** 174 primary care attenders | **Anxiety/depression:** Hospital Anxiety and Depression Scale (HADS) and sub-scales (HADS-A, HADS-D)  **Depression:** Patient Health Questionnaire (PHQ-9)  **Functioning:** WHO Disability Assessment 2.0 (WHODAS 2.0)  **PTSD:** PTSD checklist (PCL-5)  **Progress on problems for which help was sought:** Psychological Outcome Profiles (PSYCHLOPS) | N/A | **Anxiety/depression:** HADS total effect size 0.88 at 1 week (*p* < 0.001), 0.83 at 3 months; HADS-A effect size 0.76 (*p* < 0.001) at 1 week, 0.74 (*p* < 0.001) at 3 months; HADS-D effect size 0.91 (*p* < 0.001) at 1 week, 0.85 (*p* < 0.001) at 3 months  **Depression:** PHQ-9 effect size 0.87 (*p* < 0.001) at 1 week, 0.73 (*p* < 0.001) at 3 months  **Functioning:** Effect size 0.72 (*p* < 0.001) at 1 week, 0.67 (*p* < 0.001 at 3 months)  **PTSD:** Effect size 0.54 (*p* < 0.001) at 1 week, 0.63 (*p* < 0.001) at 3 months  **Progress on problems for which help was sought:** Effect size 0.34 (*p* < 0.001) at 3 months (no outcome assessment at 1-week follow-up)  **Negative effects:** Not reported |
| ***SEAR*** |  |  |  |  |  |  |  |  |  |  |
| Thailand | Common Elements Treatment Approach | Mae Sot community on Thailand-Myanmar border | Murray et al. 2014 | Displaced Burmese survivors of torture and systemic violence | CMDs: Alcohol use, anxiety, depression, PTSD | Uncontrolled before-and-after study  **Outcome assessment:** weekly monitoring (4-15 weeks) | 22 clients, 20 counsellors | **Anxiety/depression:** Hopkins Symptom Checklist (HSCL)  **Functioning:** Locally developed, gender-specific Likert scale  **PTSD:** Harvard Trauma Questionnaire (HTQ) | **Acceptability** (i.e. attrition): All clients completed treatment, though six had incomplete follow-up assessments (unavailable due to relocation or poor health)  **Fidelity:** For supervisors, review of weekly call notes between trainers and local supervisors indicated supervisors were able to maintain reasonable counsellor fidelity to the model, as evidenced by “order” and “dosing” of components and completion of documentation for pilot cases. For counsellors, review of local supervisors’ and trainers’ notes indicated “good fidelity to the model” as evidenced by movement between and completion of each component’s steps | **Depression**: 81.3% reported clinically significant change (Reliable Change Index, minimum threshold *z* > 1.96)  **Functioning:** 37.5% reported clinically significant change  **PTSD:** 68.8% reported clinically significant change |
|  |  |  | Bolton et al. 2014 | Displaced Burmese survivors of torture and systemic violence | CMDs: Alcohol use, anxiety, depression, PTSD | 2-arm randomised controlled trial  **Outcome assessment:** Four months  **Comparison group:** Waitlist | **Intervention:** 182 refugees  **Comparison:** 165 refugees | **Aggression:** Aggression Questionnaire (AQ)  **Alcohol use:** Alcohol Use Disorders Identification Test (AUDIT)  **Anxiety/depression:** Hopkins Symptom Checklist (HSCL-25)  **Functioning:** Locally developed, gender-specific Likert scale **PTSD:** Harvard Trauma Questionnaire (HTQ) | **Acceptability** (i.e. attrition): Fewer losses to follow-up in intervention arm (*n* = 34) than in control arm (*n* = 39). | **Aggression:** Adjusted effect size Cohen’s *d* = 0.58 (*p* < 0.001)  **Alcohol use:** Mean difference in pre-/post-score change between intervention and comparison group -0.03 (CI -0.44,0.50)  **Anxiety:** Adjusted effect size 0.79 (*p* < 0.001)  **Depression:** Adjusted effect size 1.16 (*p* < 0.001)  **Functioning:** Adjusted effect size 0.63 (*p* < 0.001)  **PTSD:** Adjusted effect size 1.19 (*p* < 0.001) |
|  |  |  | Murray et al. 2019a |  |  | Secondary analysis investigating possible effect of client and intervention characteristics on outcomes of randomised controlled trial (Bolton et al. 2014) | Data from 166 refugees in intervention arm (see Bolton et al. 2014) |  | N/A | No significant association between baseline client characteristics and rate of change in symptoms observed by Bolton et al. 2014 |
|  |  |  | Murray et al. 2019b |  |  | Analysis of Client Monitoring Form data to investigate fidelity and flexibility in treatment delivery during randomised controlled trial (Bolton et al. 2014) | Data from 145 treatment completers in intervention arm (Bolton et al. 2014) | N/A | **Fidelity:** 100% provider compliance on delivery of "core flow elements" of CETA, measured via provider-completed weekly Client Monitoring Form (recording up to three CETA elements, plus session duration) | N/A |
