# Supplementary material for: Lay-delivered talk therapies for adults affected by humanitarian crises in low- and middle-income countries
Source: Confl Health. 2021 Apr 23;15:30. doi: 10.1186/s13031-021-00363-8 (PMC8062937; doi:10.1186/s13031-021-00363-8)
Supplement: Supplementary file 2 — Additional file 2. “Characteristics of interventions”. [file 13031_2021_363_MOESM2_ESM.docx]

| Additional File 2: Key characteristics of interventions | | | | | | | | | |
| --- | --- | --- | --- | --- | --- | --- | --- | --- | --- |
| **Therapy type** | **Country** | **Author (Year)** | **Description of Talk Therapy** | **Dosage, Group/Individual** | **Description of Lay Workers** | **Training of Lay Workers** | **Supervision of Lay Workers** | **Description of Supervisors** | **Training of Supervisors** |
| CETA | Colombia | Bonilla-Escobar et al. 2018;  Pacichana-Quinayáz et al. 2016 | Modular, flexible transdiagnostic approach based on CBT. 9 elements: encouraging participation, psychoeducation, cognitive coping, gradual exposure (trauma memories), cognitive reprocessing, safety skills, relaxation, behavioural activation, live gradual exposure | 12-14 weekly, 1.5-hour individual sessions | **Lay psychosocial community workers**: Afro-Caribbean survivors of violence and displacement recognised as leaders and/or carers in participating communities. Minimum 5 years post-primary education and no previous mental health experience | “Apprenticeship model”: 10-day in-person training followed by weekly practice groups led by psychologist and local supervisor, then at least 1 supervised pilot case | In addition to supervising training activities, psychologist and local supervisor offered personalised supervision meetings | Psychologist and local supervisor (1 supervisor per city from local ACOPLE programme) | “Apprenticeship model”: Local supervisors received weekly Skype calls with CETA trainers (1-2 hours per week) |
|  | Thailand | Bolton et al. 2014; Murray et al. 2014, 2019a, 2019b | Modular, flexible transdiagnostic approach based on CBT, tailored to the cultural needs of the Burmese community.  9 elements: engagement, psychoeducation, anxiety management (optional), behavioural activation (optional), cognitive coping/restructuring, imaginal gradual exposure, in vivo exposure (optional), safety, Screening and Brief Intervention (SBI) for alcohol (optional) | 7-13 weekly, 1-hour, individual sessions (5-14 sessions for pilot cases in Murray et al. 2014) | **Lay counsellors**: Staff from 3 local service organisations, all Burmese refugees or members of Burmese community, literate in Burmese and with interest in mental health/counselling.  Minimum education requirement not described | “Apprenticeship model”: 10-day in-person training followed by practice groups led by local supervisors, then at least 1 supervised pilot case | In addition to supervising training activities, each local supervisor met with a small group of counsellors for 2-4 hours per week | Staff from 3 local service organisations, each in a leadership role and with minimum high school education. 1 doctor, 1 mental health counsellor, and 1 former political prisoner (with no counselling experience or advanced degree). All male Burmese refugees or members of Burmese community, bilingual in English and Burmese, and with an interest in mental health/counselling | “Apprenticeship model”: 10-day in-person CETA training followed by practice groups and additional training in supervision practices. At least 2 hours per week of supervision from the US-based CETA trainers |
| CPT | DRC | Bass et al. 2013 | Cognitive-only model (no trauma narrative) adapted for illiterate participants and those potentially exposed to ongoing violence, through: initial individual psychoeducational session, oral completion of assignments during group sessions, simplification of materials to facilitate understanding and memorisation | 11 group sessions (6-8 women per group) preceded by 1 individual session | **Psychosocial assistants:** Local NGO staff with minimum 4 years post-primary education, 1-9 years experience providing case management and supportive counselling, and prior training (5-6 days) by International Rescue Committee in case management and related topics | 2-week in-person training using locally adapted and translated manual, facilitated by trainers from the US | “Multitiered supervision system”: Direct supervision by Congolese psychosocial supervisors through weekly telephone or in-person meetings, plus in-country supervision by a clinical social worker liaising with US trainers through weekly calls | Congolese psychosocial supervisors already employed by International Rescue Committee, plus a US-trained bilingual clinical social worker as in-country supervisor | No further details |
|  | Tanzania | Greene et al. 2019 | “Nguvu” intervention involving brief CPT (6-session protocol) combined with advocacy counselling for intimate partner violence and homework activities, covering: explanation of thoughts and feelings, exploration of stuck thoughts, ABCs (becoming aware of the connection between an event and the resulting thoughts and feelings), challenging maladaptive thoughts, safety plans and relaxation training | 8 weekly two-hour sessions: 6 group CPT sessions, book-ended by 2 advocacy counselling sessions (1 individual and 1 group session). Group sessions co-facilitated in pairs | **Lay facilitators:**  Local Swahili-speaking refugee incentive workers with experience with gender-based violence programmes in the refugee camp.  Minimum education requirement not described | 9-day in-person training by an English-speaking clinical psychologist and medical anthropologist with relevant expertise, and a Swahili translator with clinical expertise. Followed by 2-day refresher training after 2 months, and 4.5-day refresher after 6 months, both conducted in Swahili by local mental health professionals | Ongoing supervision provided via phone and intermittent field visits by clinical supervisor, who observed sessions and provided feedback to facilitators through group supervision meetings. Paired delivery of group sessions also enabled peer-to-peer supervision | Swahili-speaking mental health professional hired as clinical supervisor and liaised with international investigators as needed | No further details |
| IPT | Egypt | Meffert et al. 2014 | Brief structured IPT aiming to change current relationships to improve mood symptoms. First 2 sessions work on developing “interpersonal inventory”, middle sessions work on identified focus (interpersonal disputes, role transitions, or grief), final sessions reflect on emotional and interpersonal accomplishments and goals | 6-8 individual sessions delivered twice per week | **Community therapists:** Sudanese community members fluent in English and Sudanese Arabic (oral/writing) who have previously worked with refugee populations but no prior mental health training. Personal interest and “soft” skills considered in selection process. Minimum education requirement not described | 1-week in-person training facilitated by lead investigator, followed by pilot case | Formal group supervision (twice per week) and informal supervision (daily) by lead investigator | Lead investigator, a psychiatric resident from a US teaching hospital | No further details |
| NET | Uganda | Durant 2019;  Kandah 2017 | NET, a trauma-focused therapy based on principles of prolonged exposure therapy and trauma-focused CBT (see Neuner et al. 2008, Onyut 2005, below). Delivered via peer counsellors matched by language and country of origin | Weekly sessions of individual NET (Durant 2019, 5-19 sessions;  Kandah 2017, 4-14 sessions). Maximum 2 hours per session | **Local peer counsellors:** 8 peer counsellors from 5 countries (Uganda, Rwanda, Burundi, DRC, South Sudan), working with local organisation. Minimum primary school education, shared language with target population and sufficient verbal and written English to understand training required | 6-week in-person training (minimum 72 hours total) by an interdisciplinary group of clinicians: clinical psychologist, clinical psychology doctoral student, and 4 medical students | Supervision by international clinicians through field visits and remotely via e-mail, Skype and social media. Group supervision through monthly counselling meetings arranged by the local organisation | International clinicians (clinical psychologist and clinical psychology doctoral student) | No further details |
|  |  | Ertl et al. 2011 | “KidNET”: NET adapted for youth (see Neuner et al. 2008, Onyut 2005, below) | 8 individual sessions, 3 times per week, 90-120 minutes per session | **Local lay counsellors:** Lay people from local communities with no mental health or medical background; no further details | "Intensively trained"; no further details | “Supervision meetings”; no further details | No further details | No further details |
|  |  | Neuner et al. 2008; Onyut 2005 | NET, combining aspects of Testimony Therapy and Exposure Therapy by facilitating construction of a detailed chronological account of the patient’s biography, confronting traumatic events in the process. With therapist matching where possible, based on gender, age, village of origin, and other shared factors | 4 individual sessions of NET (Onyut 2005, 4-6 sessions), typically twice per week, 1-2 hours per session | **Lay counsellors:** Rwandan and Somali refugees from the local community, literate in English and mother tongue, with minimum primary school education. Also selected for empathy and motivation | 6- to 8-week training by postdoctoral- and doctoral-level personnel, including theoretical orientation as well as expert-supervised group work and individualised training programme with private tutorials and supervised pilot cases | Weekly “case and personal supervision”; twice-monthly supervision by foreign expert trainers | Trainers were  postdoctoral- and doctoral-level personnel from German and Ugandan universities and aid organisation *vivo*; unclear which trainers were involved in supervision | No further details |
| PM+ | Pakistan | Rahman et al. 2016a; Rahman et al. 2016b | Transdiagnostic approach based on CBT, with a focus on problem-solving and behavioural techniques; includes motivational interviewing, common reactions to adversity, basic stress management, problem-solving, behavioural activation, social support networks and retaining treatment gains | 5 weekly, 90-minute individual sessions | **Lay health workers:** Minimum 12 years education with no previous clinical training or experience in counselling, social work, clinical psychology, or psychiatry | “Apprenticeship model”: 8-day in-person training programme provided by supervisors to lay health workers, followed by 3 supervised practice cases | Supervised in small groups on a weekly basis (2 hours per week) | Local mental health specialists | “Apprenticeship model": Master trainer conducted a 6-day training with local mental health specialists. Supervised (1-2 hours per month by Skype) by the master trainer |
| PST | Zimbabwe | Abas et al. 2018 | “TENDAI” PST for adherence and depression (PST-AD), delivered by adherence counsellor in HIV clinic. Depression component covers: psychoeducation; identifying and selecting problem to focus on; brainstorming, rating and selecting solutions; planning and evaluating progress | 6 weekly individual sessions: 1 50-minute and 4 30-minute therapy sessions preceded by 1 50-minute adherence session | **Adherence counsellor:** Secondary school education and 6 months of training in HIV/AIDS basic counselling, already working in primary care | No further details | Weekly meeting with psychologist to discuss caseload | Psychologist | No further details |
|  |  | Abas et al. 2016, Chibanda et al. 2017 | “Friendship Bench” PST as described in Chibanda et al. 2011, with further structuring of the intervention, particularly in terms of behavioural activation (e.g. written prompts to elicit client-generated solutions and encourage positive behaviours) | 6 individual sessions as described by Chibanda et al. 2011 | **Lay health workers** (“Grandmother health providers”), as described in Chibanda et al. 2011 | 8-day training as described in Chibanda et al. 2011, plus booster training in PST and behavioural activation facilitated by UK university and Zimbabwean diaspora organisation trainers | Regular supervision by volunteer counsellor with support from trainee psychologist interns; monthly supervision visits by psychiatrist | Volunteer counsellor and Zimbabwean psychiatrist | No further details |
|  |  | Chibanda et al. 2011 | “Friendship Bench”: Brief individual talk therapy based on PST, enhanced with activity scheduling. Delivered on a bench outside the clinic. Sessions cover: problem identification/exploration; home visit/prayer; action plan; implementation; home visit/follow-up; reinforcement | 6 weeks of 30- to 45-minute individual sessions (maximum 6 sessions) | **Lay health workers** (“Grandmother health providers”): Literate older women (mean age 58) who have lived locally for at least 15 years and are currently working as health promoters in primary care. Minimum primary school education | 8-day training run by 2 clinical psychologists, general nurse, and psychiatrist, followed by 2-day pre-testing | Daily peer-support group facilitated by 1 lay worker; weekly group supervision by clinic staff nurse; 1-hour fortnightly group supervision by clinical psychologist; 45-minute monthly group supervision by psychiatrist | Clinical psychologists, general nurse, psychiatrist | No further details |
|  |  | Chibanda et al. 2016; Munetsi et al. 2018 | “Friendship Bench” PST, a structured approach to enabling a more positive orientation toward resolving problems and improving sense of coping and control in life (see Chibanda et al. 2011, 2017; Abas et al. 2016) | Up to 6 individual sessions, generally completed within 3 weeks: 5 30- to 45-minute sessions and a single 1-hour session. Up to 6 additional text messages/phone calls for encouragement | **Lay health workers:** Older women (mean age 53) able to use a mobile phone, residing near and working in selected clinics. Mean education 10 years | 9-day manualised Friendship Bench training | Supervision provided within existing supervisory system; no further details | Trained senior health promotion officers who were part of the existing supervisory system for lay health workers | No further details |
|  |  | Chibanda et al. 2014 | Group PST tailored to post-partum period and modelled on a 7-step depression management plan previously developed for use in Zimbabwe, covering: psychoeducation, communication,  assessment of suicide risk, family involvement,  community resources, support systems, follow-up | 6 weeks of biweekly 60-minute group sessions | **Peer counsellors:** HIV-infected women with minimum secondary education who have disclosed status to partner/family, previously participated in a PMTCT programme and currently enrolled in support groups | 2-day training workshop led by 2 psychiatrists | Weekly meetings of peer counsellors to discuss their experiences, offering opportunity for peer supervision, combined with ongoing supervision by lead investigator (psychiatrist) | Zimbabwean psychiatrist | No further details. |
| THP | Pakistan | Atif et al. 2015;  Atif et al. 2016 | “Thinking Healthy Programme—Peer delivered”: CBT-based “Thinking Healthy” intervention (see Rahman 2007; Rahman et al. 2008) adapted for delivery by Peer volunteers in a mixed (individual and group) format | 7 fortnightly individual sessions and 3 monthly group sessions, over a 4-month period | **Peer volunteers:** Volunteer lay women from the community, fluent in local language, with shared sociodemographic and life experiences with the target population. Minimum 10 years education | Cascade model: 4-day classroom and 2-day field training led by non-specialist facilitators, who in turn were trained by a specialist. No further details on facilitator training | Cascade model: fortnightly group and field supervision by non-specialist facilitators, who in turn were supervised by a specialist | Non-specialist facilitators and specialist | No further details |
|  |  | Rahman 2007; Rahman et al. 2008 | “Thinking Healthy Programme”: fully manualised intervention based on CBT (specifically, "the 'here and now' problem-solving CBT approach") covering: (1) identification of unhealthy thinking styles; (2) replacement with healthy thinking; (3) homework to practice healthy thinking | 16 45-minute individual sessions organised in 5 modules: 4 weekly sessions in last month of pregnancy; 3 fortnightly sessions in the first postnatal month; then 9 monthly sessions | **Lady health workers:** Members of the local community employed in primary care mainly to provide preventive mother and child health care and education. Minimum secondary school education | 2-day workshop followed by a 1-day refresher after 3 months | Monthly half-day group supervision sessions led jointly by mental health professional and public health expert | Psychiatrist and mother-and-child expert | No further details |
