# Supplementary material for: Lay-delivered talk therapies for adults affected by humanitarian crises in low- and middle-income countries
Source: Confl Health. 2021 Apr 23;15:30. doi: 10.1186/s13031-021-00363-8 (PMC8062937; doi:10.1186/s13031-021-00363-8)
Supplement: Supplementary file 3 — Additional file 3. “Results of EPHPP Assessment”. [file 13031_2021_363_MOESM3_ESM.docx]

| Additional File 3: Results of EPHPP* Assessment | | | | | | | |
| --- | --- | --- | --- | --- | --- | --- | --- |
| **Author (Year)** | **Selection Bias** | **Study Design** | **Confounders** | **Blinding** | **Data Collection** | **Attrition** | **Overall** |
| Abas et al. 2016 | Moderate | Weak | Weak | Weak | Weak | Weak | **Weak** |
| Abas et al. 2018 | Weak | Strong | Weak | Moderate | Moderate | Moderate | **Weak** |
| Bass et al. 2013 | Strong | Moderate | Strong | Moderate | Strong | Weak | **Moderate** |
| Bolton et al. 2014 | Moderate | Strong | Strong | Moderate | Strong | Moderate | **Strong** |
| Bonilla-Escobar et al. 2018 | Moderate | Strong | Strong | Moderate | Strong | Moderate | **Strong** |
| Chibanda et al. 2011 | Moderate | Moderate | Weak | Weak | Strong | Strong | **Weak** |
| Chibanda et al. 2014 | Moderate | Moderate | Weak | Moderate | Strong | Moderate | **Moderate** |
| Chibanda et al. 2016 | Strong | Strong | Strong | Strong | Strong | Strong | **Strong** |
| Durant 2019 | Weak | Moderate | Weak | Weak | Weak | Weak | **Weak** |
| Ertl et al. 2011 | Strong | Strong | Strong | Moderate | Strong | Strong | **Strong** |
| Greene et al. 2019 | Moderate | Weak | Weak | Moderate | Strong | Weak | **Weak** |
| Kandah 2017 | Weak | Moderate | Weak | Weak | Weak | Moderate | **Weak** |
| Meffert et al. 2014 | Moderate | Strong | Weak | Weak | Strong | Strong | **Weak** |
| Murray et al. 2014 | Weak | Moderate | Weak | Weak | Strong | Moderate | **Weak** |
| Murray et al. 2019b | Moderate | Weak | Weak | Moderate | Strong | Strong | **Weak** |
| Neuner et al. 2008 | Moderate | Strong | Strong | Moderate | Strong | Weak | **Moderate** |
| Rahman 2007 | Weak | Weak | Weak | Weak | Weak | Weak | **Weak** |
| Rahman et al. 2008 | Strong | Strong | Strong | Strong | Strong | Strong | **Strong** |
| Rahman et al. 2016a | Moderate | Strong | Weak | Moderate | Weak | Weak | **Weak** |
| Rahman et al. 2016b | Moderate | Strong | Strong | Moderate | Strong | Strong | **Strong** |
| **EPHPP: Effective Public Health Practice Project (EPHPP) Quality Assessment Tool for Quantitative Studies* | | | | | | | |
