# Supplementary material for: Lay-delivered talk therapies for adults affected by humanitarian crises in low- and middle-income countries
Source: Confl Health. 2021 Apr 23;15:30. doi: 10.1186/s13031-021-00363-8 (PMC8062937; doi:10.1186/s13031-021-00363-8)
Supplement: Supplementary file 4 — Additional file 4. “Results of CASP Assessment”. [file 13031_2021_363_MOESM4_ESM.docx]

| Additional File 4: Results of CASP Assessment* | | | | | | | | | | |
| --- | --- | --- | --- | --- | --- | --- | --- | --- | --- | --- |
| **Author (Year)** | **Clear statement of aims?** | **Qualitative methodology appropriate?** | **Research design appropriate?** | **Recruitment strategy appropriate?** | **Data collection addressed the issue?** | **Researcher-participant relationship considered?** | **Ethical issues considered?** | **Rigorous data analysis?** | **Clear statement of findings?** | **Valuable research?** |
| Abas et al. 2016 | Yes | Yes | Yes | No | Yes | No | Yes | Yes | Yes | Yes |
| Abas et al. 2018 | Yes | Yes | Yes | No | Yes | No | Yes | Yes | Yes | Yes |
| Atif et al. 2016 | Yes | Yes | Yes | Yes | Yes | Yes | Yes | Yes | Yes | Yes |
| Chibanda et al. 2011 | No | Yes | No | No | Yes | No | Yes | No | Yes | Yes |
| Chibanda et al. 2017 | Yes | Yes | Yes | Yes | Yes | No | Yes | Yes | Yes | Yes |
| Greene et al. 2019 | Yes | Yes | Yes | Yes | Yes | No | Yes | No | Yes | Yes |
| Pacichana-Quinayáz et al. 2016 | Yes | Yes | Yes | No | Yes | No | Yes | Yes | Yes | Yes |
| Rahman et al. 2016a | No | Yes | No | No | No | No | Yes | No | Yes | Yes |
| **CASP: Critical Appraisal Skills Programme Qualitative Researcher Checklist* | | | | | | | | | | |
