# Supplementary material for: Lay-delivered talk therapies for adults affected by humanitarian crises in low- and middle-income countries
Source: Confl Health. 2021 Apr 23;15:30. doi: 10.1186/s13031-021-00363-8 (PMC8062937; doi:10.1186/s13031-021-00363-8)
Supplement: Supplementary file 5 — Additional file 5. “Search Terms for Ovid MEDLINE(R)”. [file 13031_2021_363_MOESM5_ESM.docx]

**Additional File 5: Search Terms for Ovid MEDLINE(R)**

**(1) Low-and Middle-Income Country**

1. Developing Countr*.sh,kf.

2. (Africa or Asia or Caribbean or West Indies or South America or Latin America or Central America).hw,kf,ti,ab.

3. (Afghanistan or Albania or Algeria or Angola or Antigua or Barbuda or Argentina or Armenia or Armenian or Aruba or Azerbaijan or Bahrain or Bangladesh or Barbados or Benin or Byelarus or Byelorussian or Belarus or Belorussian or Belorussia or Belize or Bhutan or Bolivia or Bosnia or Herzegovina or Hercegovina or Botswana or Brazil or Bulgaria or Burkina Faso or Burkina Fasso or Upper Volta or Burundi or Urundi or Cambodia or Khmer Republic or Kampuchea or Cameroon or Cameroons or Cameron or Camerons or Cape Verde or Central African Republic or Chad or Chile or China or Colombia or Comoros or Comoro Islands or Comores or Mayotte or Congo or Zaire or Costa Rica or Cote d'Ivoire or Ivory Coast or Croatia or Cuba or Cyprus or Czechoslovakia or Czech Republic or Slovakia or Slovak Republic or Djibouti or French Somaliland or Dominica or Dominican Republic or East Timor or East Timur or Timor Leste or Ecuador or Egypt or United Arab Republic or El Salvador or Eritrea or Estonia or Ethiopia or Fiji or Gabon or Gabonese Republic or Gambia or Gaza or Georgia Republic or Georgian Republic or Ghana or Gold Coast or Greece or Grenada or Guatemala or Guinea or Guam or Guiana or Guyana or Haiti or Honduras or Hungary or India or Maldives or Indonesia or Iran or Iraq or Isle of Man or Jamaica or Jordan or Kazakhstan or Kazakh or Kenya or Kiribati or Korea or Kosovo or Kyrgyzstan or Kirghizia or Kyrgyz Republic or Kirghiz or Kirgizstan or Lao PDR or Laos or Latvia or Lebanon or Lesotho or Basutoland or Liberia or Libya or Lithuania or Macedonia or Madagascar or Malagasy Republic or Malaysia or Malaya or Malay or Sabah or Sarawak or Malawi or Nyasaland or Mali or Malta or Marshall Islands or Mauritania or Mauritius or Agalega Islands or Mexico or Micronesia or Middle East or Moldova or Moldovia or Moldovian or Mongolia or Montenegro or Morocco or Ifni or Mozambique or Myanmar or Myanma or Burma or Namibia or Nepal or Netherlands Antilles or New Caledonia or Nicaragua or Niger or Nigeria or Northern Mariana Islands or Oman or Muscat or Pakistan or Palau or Palestine or Panama or Paraguay or Peru or Philippines or Philipines or Phillipines or Phillippines or Poland or Portugal or Puerto Rico or Romania or Rumania or Roumania or Russia or Russian or Rwanda or Ruanda or Saint Kitts or St Kitts or Nevis or Saint Lucia or St Lucia or Saint Vincent or St Vincent or Grenadines or Samoa or Samoan Islands or Navigator Island or Navigator Islands or Sao Tome or Saudi Arabia or Senegal or Serbia or Montenegro or Seychelles or Sierra Leone or Slovenia or Sri Lanka or Ceylon or Solomon Islands or Somalia or Sudan or Suriname or Surinam or Swaziland or Syria or Tajikistan or Tadzhikistan or Tadjikistan or Tadzhik or Tanzania or Thailand or Togo or Togolese Republic or Tonga or Trinidad or Tobago or Tunisia or Turkey or Turkmenistan or Turkmen or Uganda or Ukraine or Uruguay or USSR or Soviet Union or Union of Soviet Socialist Republics or Uzbekistan or Uzbek or Vanuatu or New Hebrides or Venezuela or Vietnam or Viet Nam or West Bank or Yemen or Yugoslavia or Zambia or Zimbabwe or Rhodesia).hw,kf,ti,ab,cp.

4. ((developing or least-developed or less-developed or under-developed or underdeveloped or low-income or middle-income or transitional) adj3 (countr* or nation? or world or econom*)).ab,ti.

5. (LAMIC? or LIC? LMIC? or MIC? or UMIC?).ab,ti.

6. ((LAMI or LI or LMI or MI or UMI) adj3 countr*).ab,ti.

7. 1 or 2 or 3 or 4 or 5 or 6

**(2) Talking Therapy**

8. exp Psychotherapy/

9. exp Counseling/

10. ((brief or low-intensity or scalable) adj2 (intervention? or treatment?)).mp.

11. ((exposure or cognitive* or behavio?r* or group or inter-personal or interpersonal or narrative or problem or psychosocial or talk* or commitment or testimony or motivational or contingency) adj2 (therap* or intervention? or treatment? or program* or package? or training?)).mp.

12. (thinking healthy or problem-management or psychological or psycho-therap* or psychotherap* or counsel*).mp.

13. (CBT or CPT or IPT or IPT-G or NET or PST or ACT or PM? or THP).mp.

14. 8 or 9 or 10 or 11 or 12 or 13

**(3) Lay Worker**

15. exp Allied Health Personnel/

16. Community Health Worker?.mp.

17. Nurse* Aide?.mp.

18. Psychiatric Aide?.mp.

19. Caregiver?.mp.

20. Voluntary worker?.mp.

21. Volunteer?.mp.

22. Community network?.mp.

23. ((community or lay or voluntary or volunteer? or untrained or trained or unlicen?ed or nonprofessional? or non-professional? or peer?) adj3 (worker? or visitor? or attendant? or aide? or support* or person* or helper? or care* or consultant? or advisor? or counsel* or assistant? or staff or therapist)).mp.

24. (peer* adj3 (work* or counsel* or deliver*)).mp.

25. (paraprofessional? or para-professional?).mp.

26. (allied health adj2 (worker? or professional? or personnel)).mp.

27. Support worker?.mp.

28. (non-specialist? or nonspecialist?).mp.

29. Specially-trained.mp.

30. Barefoot doctor?.mp.

31. ((nurs* or psychiatric) adj3 (aid* or assistant* or attendant*)).mp.

32. (informal adj3 care*).mp.

33. ((self-help or support) adj3 group?).mp.

34. Teacher?.mp.

35. School staff.mp.

36. Trainer?.mp.

37. (Village adj3 worker?).mp.

38. CHW?.mp.

39. VHW?.mp.

40. PSW?.mp.

41. (task* adj3 (shar* or shift*)).mp.

42. ((collaborative or stepped) adj2 care).mp.

43. (communit* adj3 (based or intervention* or network*or service?)).mp.

44. 15 or 16 or 17 or 18 or 19 or 20 or 21 or 22 or 23 or 24 or 25 or 26 or 27 or 28 or 29 or 30 or 31 or 32 or 33 or 34 or 35 or 36 or 37 or 38 or 39 or 40 or 41 or 42 or 43

**(4) Common Mental Disorders**

45. (Common mental disorder? or common mental health disorder? or CMD? or CMHD?).ab,ti.

***Mood [affective] disorders (F32-F39)***

46. exp Mood Disorders/

47. Depress*.ab,ti.

48. Mood disorder?.ab,ti.

49. (Affective adj2 disorder?).ab,ti.

50. Cyclothymi*.ab,ti.

51. Cycloid.ab,ti.

52. Dysthymi*.ab,ti.

***Neurotic, stress-related and somatoform disorders (F40-F48)***

53. exp Anxiety Disorders/

54. exp "Trauma and Stressor Related Disorders"/

55. exp Dissociative disorders/

56. exp Somatoform Disorders/

57. $phobi*.ab,ti.

58. Anxiety.ab,ti.

59. Anxious.ab,ti.

60. (panic adj2 (disorder? or attack? or state?)).ab,ti.

61. Obsessi*.ab,ti.

62. Compulsi*.ab,ti.

63. OCD.ab,ti.

64. Neurotic.ab,ti.

65. Neuros?s.ab,ti.

66. ((psychological* or emotional* or psychic) adj3 (distress* or stress* or trauma* or shock* or cris$s)).ab,ti.

67. ((acute* or severe*) adj3 (stress* or distress*)).ab,ti.

68. ((post-trauma* or posttrauma*) adj2 (stress or distress or disorder?)).ab,ti.

69. PTSD.ab,ti.

70. Fatigue syndrome.ab,ti.

71. ((cris?s or grief) adj3 (reaction? or state?)).ab,ti.

72. Dissociati*.ab,ti.

73. Hysteri*.ab,ti.

74. (conversion adj2 (disorder? or reaction?)).ab,ti.

75. Multiple personality.ab,ti.

76. Somatoform.ab,ti.

77. Somati?ation.ab,ti.

78. Psychosomatic.ab,ti.

79. Psychogenic.ab,ti.

80. Hypochondria*.ab,ti.

81. Dysmorphi*.ab,ti.

82. Neurocirculatory asthenia.ab,ti.

83. Neurastheni*.ab,ti.

84. ((depersonali?ation or dereali?ation) adj2 disorder?).ab,ti.

***Mental and behavioural disorders due to use of psychoactive substances (F10-F19)***

85. exp Substance-Related Disorders/

86. exp Psychotropic Drugs/

87. ((intoxication or harmful or hazardous or dependen* or withdrawal or flashback* or psychotic or pschosis or amnesi?c or disorder? or illness* or $use or addict*) adj5 (alcohol or drinking or opioid? or cannabinoid? or cannabis or marijuana or sedative? or hypnotic? or cocaine or stimulant? or hallucinogen? or tobacco or (volatile adj2 solvent?) or psychoactive or drug? or substance? or inhalant? or amphetamine?)).ab,ti.

88. (SUD? or AUD?).ab,ti.

89. ((acute or pathological) adj3 (drunkenness or intoxication)).ab,ti.

90. Alcoholi*.ab,ti.

91. ((amnesi?c or dependen* or Korsakov or Wernicke*) adj3 (psychosis or disease or syndrome)).ab,ti.

92. 45 or 46 or 47 or 48 or 49 or 50 or 51 or 52 or 53 or 54 or 55 or 56 or 57 or 58 or 59 or 60 or 61 or 62 or 63 or 64 or 65 or 66 or 67 or 68 or 69 or 70 or 71 or 72 or 73 or 74 or 75 or 76 or 77 or 78 or 79 or 80 or 81 or 82 or 83 or 84 or 85 or 86 or 87 or 88 or 89 or 90 or 91

**(5) Humanitarian Crisis**

93. exp Disasters/

94. exp Violence/

95. exp Warfare/

96. Abduct*.mp.

97. Abus*.mp.

98. Adversit*.mp.

99. Asylum-seek*.mp.

100. Avalanche?.mp.

101. Combat?.mp.

102. Conflict?.mp.

103. Cris?s.mp.

104. Cyclon*.mp.

105. Disaster?.mp.

106. Displaced.mp.

107. Drought?.mp.

108. Earthquake?.mp.

109. Emergenc*.mp.

110. Epidemic*.mp.

111. Erupt*.mp.

112. Evacu*.mp.

113. Explod*.mp.

114. Explosion*.mp.

115. Famine?.mp.

116. Fire?.mp.

117. Flood*.mp.

118. Genocide?.mp.

119. Humanitarian.mp.

120. Hunger.mp.

121. Hurricane?.mp.

122. IDP?.mp.

123. Landslide?.mp.

124. Mass casualty incident?.mp.

125. Migrant?.mp.

126. Outbreak?.mp.

127. Rape?.mp.

128. Refugee?.mp.

129. Relief work?.mp.

130. Rescue work?.mp.

131. Soldier?.mp.

132. Starv*.mp.

133. Storm?.mp.

134. Survivor?.mp.

135. Terror*.mp.

136. Tortur*.mp.

137. Tsunami?.mp.

138. Typhoon?.mp.

139. Pandemic?.mp.

140. Veteran?.mp.

141. Victim?.mp.

142. Violen*.mp.

143. Volcan*.mp.

144. War?.mp.

145. Warfare.mp.

146. Wave?.mp.

147. 93 or 94 or 95 or 96 or 97 or 98 or 99 or 100 or 101 or 102 or 103 or 104 or 105 or 106 or 107 or 108 or 109 or 110 or 111 or 112 or 113 or 114 or 115 or 116 or 117 or 118 or 119 or 120 or 121 or 122 or 123 or 124 or 125 or 126 or 127 or 128 or 129 or 130 or 131 or 132 or 133 or 134 or 135 or 136 or 137 or 138 or 139 or 140 or 141 or 142 or 143 or 144 or 145 or 146
